# Hematite α-Fe_2_O_3_ nanorods and laser-induced graphene for sustainable chemiresistive sensing of 1-butanol at room temperature

**DOI:** 10.1039/d5na00609k

**Published:** 2025-11-05

**Authors:** Mintesinot Tamiru Mengistu, Richard Murray, Alida Russo, Cathal Larrigy, Daniela Iacopino, Colin Fitzpatrick, Michael Nolan, Aidan J. Quinn.

**Affiliations:** a Tyndall National Institute, University College Cork Lee Maltings, Dyke Parade Cork T12 R5CP Ireland aidan.quinn@tyndall.ie; b Dept of Electronic & Computer Engineering, University of Limerick Limerick V94 T9PX Ireland

## Abstract

Volatile organic compounds (VOCs) present in workplace and domestic settings present risks to human health, *e.g.*, 1-butanol concentrations >100 ppm can cause central nervous system depression and respiratory/skin irritation. Traditional chemiresistive metal-oxide gas sensor platforms frequently rely on noble metal contact electrodes (Au,Pt) and high-temperature operation (200–600 °C), increasing cost and environmental footprint impacts. Consequently, there is an urgent need for sustainable and affordable materials for chemiresistive gas sensors that can operate at room temperature. Our approach combines hematite (α-Fe_2_O_3_) nanorods, synthesized *via* a low-impact co-precipitation method, with 3D porous laser-induced graphene (LIG) electrodes for room-temperature chemiresistive sensing of VOCs. Relative humidity (RH) plays a key role in charge transport through these LIG-contacted α-Fe_2_O_3_ nanorod assemblies, with baseline device resistance *R*_0_ decreasing quasi-exponentially with increasing humidity. Device resistance increases upon exposure to 1-butanol, with resistance response Δ*R*/*R*_0_ ∼ 185 ± 25% (*n* = 8) to 100 ppm 1-butanol at ∼55% RH, with 50–300 ppm linear range and limit of detection, LOD = 36 ± 11 ppm. Device response, Δ*R*/*R*_0_, increases with increasing relative humidity from ∼20–60% RH, highlighting the key role of the hydrated α-Fe_2_O_3_ surfaces on the sensing mechanism. Measured response values represent a ∼10-fold improvement in sensitivity *vs.* reported room-temperature performance for devices based on α-Fe_2_O_3_ nanocubes. Further, the estimated cumulative energy demand (CED) for the α-Fe_2_O_3_ nanorod active nanomaterial is ∼1000 times lower than reported data for devices with comparable sensitivity, which employed α-Fe_2_O_3_ nanocubes and reduced graphene oxide hybrids. Estimated CED values for the 3-D porous LIG electrodes also show orders of magnitude reduction *vs.* values for conventional metal contact electrodes. Finally, we show that the response time constants of these LIG-contacted α-Fe_2_O_3_ nanorod devices can be used together with chemiresistive Δ*R*/*R*_0_ response for effective discrimination of 1-butanol *vs.* other short-chain alcohols (methanol, ethanol, 2-propanol) and non-polar VOCs (acetone, toluene, hexane).

## Abstract 2

Hematite α-Fe_2_O_3_ nanorod assembly contacted with porous 3D laser-induced graphene (LIG) electrodes for chemiresistive sensing of 1-butanol at room-temperature.

## Graphical abstract

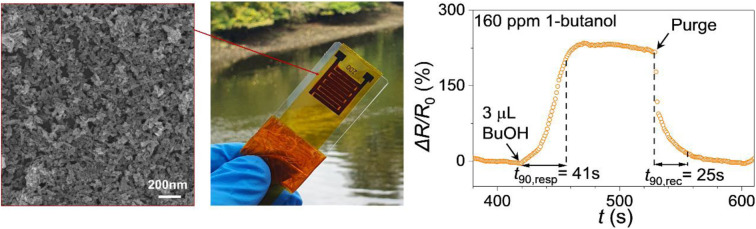

## 1 Introduction

Volatile organic compounds (VOCs) are present in our daily lives in both workplace and domestic settings and can present risks to human health. 1-Butanol (also referred to as *n*-butanol) is a VOC commonly used in varnish, plasticizers, cosmetics, detergent organic synthesis intermediates, and extractants. However, exposure to >100 ppm 1-butanol can result in severe health issues including nervous system depression, respiratory irritation, headache, dizziness, drowsiness, dermatitis and skin irritation.^1^ In addition, 1-butanol is flammable and can form an explosive mixture with air at elevated concentrations (11.5%). Accordingly, the Occupational Safety and Health Administration (OSHA) and the National Institute for Occupational Safety and Health (NIOSH) have set safety standards of 100 and 50 ppm, respectively, for 1-butanol in the workplace.^2,3^ Given the potential negative effects of 1-butanol and its excessive emissions, there is a pressing need for affordable, reliable, rapid and sensitive detection methods.

Owing to their good sensitivity and rapid response times, metal-oxide-semiconductor (MOS) nanomaterials have attracted significant interest as active materials for chemiresistive sensing of hazardous or poisonous gases.^4,5^ MOS nanomaterials including SnO_2_, ZnO, WO_3_, TiO_2_, Co_3_O_4_, α-Fe_2_O_3_, CuO, NiO, have been widely employed in gas-sensing applications.^6–18^ Among these metal oxide nanomaterials, α-Fe_2_O_3_ has attracted significant attention for 1-butanol sensing because of its high chemical stability, low manufacturing costs, and abundance. Table S1 provides an overview of previous studies on α-Fe_2_O_3_ nanomaterials for chemiresistive sensing of 1-butanol. Most studies report high operating temperatures (160–300 °C), where the sensor resistance measured following exposure to 1-butanol vapor (*R*_VOC_) was significantly lower than the ambient atmosphere value (*R*_0_). Resistance ratios in the range *R*_0_/*R*_VOC_ ∼1–50 were reported for 100 ppm 1-butanol concentrations.

**Scheme 1:**
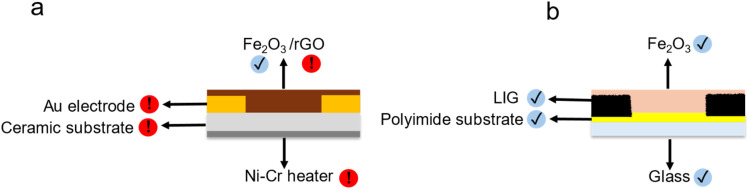
Comparative Illustration of (a) conventional and (b) LIG-chemiresistive sensor configurations highlighting material choices and sustainability.

However, high operating temperatures necessitate use of an integrated heating element and thermally-stable materials for the substrate, *e.g.* alumina or ceramic, and also the contact electrodes, *e.g.*, gold or platinum-group metals; see Scheme 1a below. The associated constraints around materials selection and manufacturing processes increase both the sensor cost and the environmental footprint impacts,^19^ including global warming potential, and resource depletion. These thermal stability constraints would also apply if further anneal steps were required following deposition of the active nanomaterial on the contact electrodes.^6^

It is challenging to perform a comprehensive, quantitative “Cradle to Grave” Life Cycle Assessment (LCA) for emerging research nanomaterials and fabrication processes at low Technology Readiness Levels due to the lack of available and/or standardized data.^20^ Thus, streamlined sustainability assessments often use comparative approaches to identify “hotspots” which can dominate the overall environmental footprint. Cumulative Energy Demand (CED), a proxy for Global Warming Potential, is a useful metric, given the strong correlation with other environmental footprint impacts.^21^

Table 1 provides rough comparative estimates of the cumulative energy demand for the source materials (active sensing nanomaterial, contact electrodes, and substrate) as well as the electricity consumption during laboratory-scale contact electrode fabrication for a range of sensors based on iron oxide (FeO_*x*_) MOS nanomaterials. Hotspots are highlighted in orange/red (Table 1 and Scheme 1a). We note that these estimates are likely to represent lower bounds for the contribution to the total CED, since not all process fabrication steps are accounted for.

**Table 1:** Estimates for Cumulative Energy Demand (CED) and comparative sustainability analysis of chemiresistive iron-oxide based sensors for detection of 1-butanol, comprising active sensing nanomaterials, electrode source materials, electrode deposition methods, and chemiresistive response to 100 ppm 1-butanol

Operating temperature	Active sensing nanomaterial	CED (MJ kg^−1^)	Contact electrode source material	CED (MJ kg^−1^)	Cost	Electrode fabrication method	Electricity demand (MJ per coupon)	Substrate material	CED (MJ kg^−1^)	Resistive response	Ref.
160°C	Fe_2_O_3_	∼200^a^	Gold	200 000^b^	High	Physical vapor deposition (PVD)	∼4.5^c^	Sintered ceramic	∼80–1800^d^	High: *R*_0_/*R*_VOC_ ∼ 800%	15
Room temp	Fe_2_O_3_	∼200^a^								Low: Δ*R*/*R*_0_ ∼ −13%	6
	Fe_2_O_3_/rGO	rGO: ∼21 000–69 000^e^								Medium: Δ*R*/*R*_0_ ∼ −170%	
	Fe_2_O_3_	∼200^a^	Polyimide	∼200^f^	Medium	Laser graphitization	∼0.01^g^	Glass	∼40^h^	Medium: Δ*R*/*R*_0_ = 185 ± 25%	This work

a Life Cycle Inventory of a range of synthesis methods for Fe_2_O_3_ and FeO_*x*_ nanoparticles, 20–200 MJ kg^−1^ (ref. 22).

b Energy Consumption values from life cycle inventory for production of bulk gold, reported in GJ per tonne (ref. 23).

c Estimates for electricity consumption based on reported data (12 650 Wh, 45.5 MJ) for one deposition run comprising lab-scale sputtering of a metal target, TiAl, ref. 24, coating a substrate of area 25 cm^2^. Calculation here assumes 10 electrode coupons produced, each 2.5 cm^2^.

d Low value (∼80 MJ kg^−1^) based on reported embodied energy data for alumina production (50–55 MJ kg^−1^) and forming (25–28 MJ kg^−1^) from ref. 25. High value estimated from reported data for lab-scale fabrication of sintered cm-scale Al_2_O_3_ ceramic tubes, ref. 26.

e Life cycle inventory for reduced graphene oxide (rGO), ref. 27.

f Embodied energy value for polyimide (∼170–195 MJ kg^−1^) from ref. 28.

g Estimate based on measured power electricity consumption (160 W × 60 s = 9.6 kJ) during fabrication of a 3 cm^2^ rectangular LIG electrode using 10.6 µm CO_2_ laser (∼3 W average laser power for LIG formation). Majority of electricity consumption related to extract and exhaust filtering system.

h Estimate based on reported values for production (27–30 MJ kg^−1^) and moulding (∼9 MJ kg^−1^) of borosilicate glass, ref. 25.

Scheme 1b illustrates our approach to addressing environmental footprint hotspots associated with nanomaterial synthesis and fabrication of chemi-resistive sensors for room-temperature detection of 1-butanol, see Discussion section below. Briefly, our approach focuses on combining α-Fe_2_O_3_ nanorods, synthesized *via* a low-impact co-precipitation method, with laser-induced graphene contact electrodes. Laser-induced graphene (LIG) is a highly porous three-dimensional conductive carbon network formed by lasing an appropriate polymer substrate, discovered in 2014 by Lin, Tour and co-workers.^29^ We have recently demonstrated chemiresistive sensing of methanol at room-temperature using LIG electrodes with low loadings of SnO mesoflower active materials.^30^ Here we report on chemiresistive sensing of 1-butanol and other VOCs at room temperature using resource-efficient, LIG-contacted α-Fe_2_O_3_ nanorod devices. We investigate the key influence of relative humidity on device performance and identify measured parameters that can be used as inputs to simple machine learning models to improve device selectivity.

## 2 Experimental

### 2.1 Materials

Iron(iii) nitrate nonahydrate (Fe (NO_3_)_3_·9H_2_O, 99.0%) and sodium hydroxide (NaOH) was supplied by Sigma-Aldrich (Shanghai, China). Polyimide tape (0.07 mm thick, silicone adhesive backing) was purchased from Sigma-Aldrich. Isopropyl alcohol (99.5%, Merck), acetone (99.5%, Merck), hexane (99.8%, Merck), 1-butanol (99.8%, Merck), methanol (99.8%, Merck), ethanol (99.9%, Merck), and toluene (99.8%, Merck) were used as the analytes. All chemicals were used as received without purification. Deionized (DI) water with electrical resistivity 18.2 MΩ cm was used for all aqueous solutions.

### 2.2 Preparation of α-Fe_2_O_3_ nanorods

Co-precipitation methods were used to synthesize α-Fe_2_O_3_ nanorods. 0.05 M Fe(NO_3_)_3_·9H_2_O was stirred in deionized (DI) water for 30 min at room temperature. NaOH in DI water (1 M, 25 mL) was slowly added to the iron nitrate solution until the pH reached 11, and the mixture was stirred for 3 h at 80 °C. The precipitate was allowed to age overnight and then centrifuged with DI water followed by ethanol. The powder was then dried in an oven at 60 °C for 6 h, crushed using a mortar, and calcined in a muffle furnace at a set temperature (400, 450, 500, 550, or 600 °C) for 3 h to obtain α-Fe_2_O_3_ nanorods.

### 2.3 Preparation of laser-induced graphene (LIG) electrodes

**Fig. 1:**
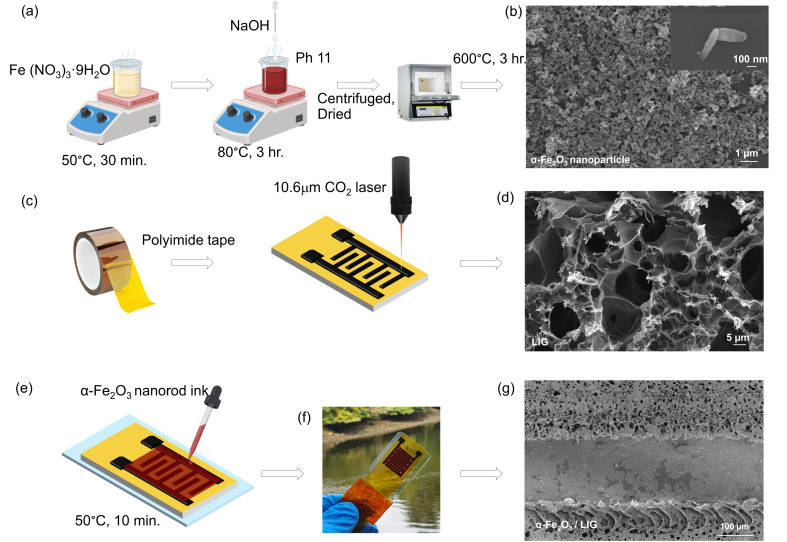
(a) Schematic illustration of the synthesis procedure for α-Fe_2_O_3_ nanorods. (b) Scanning electron microscopy (SEM) data for α-Fe_2_O_3_ nanorod assembly drop-deposited on Si (001) substrate. Inset: high-magnification SEM showing individual nanorods (c) schematic of LIG electrode fabrication (d) SEM data showing 3D porous LIG (e and f) schematic and photograph of α-Fe_2_O_3_ nanorods/LIG sensor. (g) Low-magnification SEM showing microporous assembly of α-Fe_2_O_3_ nanorods between interdigitated LIG electrodes.

Interdigitated LIG electrodes were fabricated using a 10.6 µm CO_2_ laser (Universal Laser System PLS 4.75), as depicted in Fig. 1b. In our previous work, we found that an average laser power ranging from 2.4–3.9 W with a scan speed ranging from to 280–440 mm s^−1^ yielded LIG electrodes with low sheet resistance and a low defect density.^31^ For this study, we employed an average laser power of 3 W, scan speed ∼350 mm s^−1^, and lens-sample separation of 0.51 mm. The interdigitated electrodes were designed using PowerPoint, with interelectrode gaps ∼180–200 µm (SI, Fig. S1b). For each device, the electrode area, including the gaps, was 1.6 cm^2^.

### 2.4 Preparation of LIG-contacted α-Fe_2_O_3_ nanorod assemblies

Two milligrams of α-Fe_2_O_3_ nanorods was dispersed in isopropyl alcohol (IPA, 500 µL) and ultrasonicated for 1 h to obtain a homogeneous slurry. The slurry was drop-cast onto previously prepared interdigitated LIG electrodes and heated at 50 °C for 10 min on a hot plate to evaporate the solvent. Finally, fabricated devices were stored overnight under ambient conditions. In this study, 33 sensor devices were fabricated and evaluated. One batch of synthesized α-Fe_2_O_3_ nanorods was used to prepare 23 devices (D1–D23), used to study device response to different VOCs in humidified nitrogen environments. Five devices (D24–D27, D32) were fabricated as a separate batch using identical procedures to examine device response to 1-butanol in humidified air environments. Five additional devices (D28–D31, D33) were fabricated using α-Fe_2_O_3_ nanorods synthesized at a range of calcination temperatures (400 °C < *T*_calc_ < 650 °C) to investigate the effect of *T*_calc_ on device response to 1-butanol. All devices used the same LIG electrode geometry and test conditions unless specified otherwise.

### 2.5 Characterization

Raman spectra of α-Fe_2_O_3_ and LIG were obtained using a Horiba XploRA Raman microscope using 532 nm excitation for LIG samples and 750 nm excitation for α-Fe_2_O_3_ samples, 10× objective, 30 s acquisition, one accumulation, and 10% power. Powder X-ray diffraction (PANalytical X'pert PRO, copper anode, *K*_1_ = 0.15406 nm, *K*_2_ = 0.15444 nm) was used to determine the phase composition for 2*θ* values in the range 20–80°. Surface morphology was investigated using a Zeiss Supra scanning electron microscope (SEM) at an accelerating voltage of 10 kV. Energy dispersive X-ray analysis (EDX) data and SEM-EDX maps were acquired using an Oxford X-Max 50 detector. Optical absorption spectra for α-Fe_2_O_3_ nanorods in isopropanol solutions were acquired using an Agilent/HP 8453 UV-Vis spectrophotometer over a wavelength range of 200–1100 nm.

### 2.6 Gas sensor measurements

The response of the devices to the specific VOCs was evaluated using a custom-made gas-sensing setup,^30^ using humidified nitrogen or humidified air environments. An 800 mL test chamber that could accommodate up to four devices simultaneously was used to assess the performance of the α-Fe_2_O_3_ nanorod sensors (Fig. S2). A 3D-printed polylactic acid lid with four slots was used to mount the sensor devices, which were externally connected to a multichannel multimeter (Keithley DAQ 6510-7700) *via* shielded coaxial cables. Devices were measured simultaneously at ambient laboratory temperature (∼18 °C) *via* multiplexing with 5 averaged readings per measurement (number of power line cycles, NPLC = 5), time interval between measurements: 100 ms. The multimeter voltage bias was verified as 0.4 V DC with a 0.25 V amplitude AC modulation (50 Hz).

The lid featured three holes: an inlet for purging with humidified nitrogen or humidified air (oil-free compressed air), an exhaust port for the purge gas, and a separate port for analyte injection using a microsyringe (Hamilton, 10 µL). The relative humidity (%RH) was maintained within a range of 55 ± 5% RH, intentionally created by passing dry nitrogen through a water bubbler. The test jar was positioned on a hot plate (80 °C) to allow rapid evaporation of the injected analyte. As reported previously,^30^ the gaseous phase concentration (ppm) of each analyte, corresponding to evaporation of injected liquid-phase aliquots was measured using a photoionization detector (Tiger PID, 11.7 eV lamp) standardized against a reference gas (100 ppm isobutylene in balance air) measured 3 minutes after solvent addition (see Fig. S3).

Measurements in standard humidity environments were performed in sealed centrifuge tubes fitted with a customised 3D-printed lid to facilitate appropriate device mounting. Saturated standard salt solutions were used to achieve the desired relative humidity values: sodium hydroxide (NaOH, 7.5% RH), magnesium chloride (MgCl_2_, 33% RH), sodium bromide (NaBr, 59% RH), and potassium chloride (KCl, 85% RH).^32^

### 2.7 Data analysis

The sensitivity of each sensor device was calculated from measured response values, Δ*R*/*R*_0_ = (*R*_VOC_ − *R*_0_)/*R*_0_, where *R*_0_ and *R*_VOC_ represent the sensor resistance values in humidified nitrogen (or air) and following injection of the VOC analyte, respectively. Baseline curve subtraction for Δ*R*/*R*_0_ data was performed using Origin's Peak Analyzer function. The baseline curve for each device measurement was initially established by identifying anchor points through the second derivative zero-crossing method. Anchor points were then manually adjusted to effectively account for baseline drift without distorting the signal peaks, ensuring a more precise analysis of the sensor response, see Fig. S10d and e. The *t*_90_ response and recovery times were defined as the times required for the sensor to reach 90% of the total resistance change after exposure to the target VOC and purging with humidified nitrogen (or air), respectively.

### 2.8 Random resistor network simulations and machine learning

Simulations of random resistor networks were performed using the Simulink Toolbox in MATLAB (R2022a). Rectangular resistor networks were constructed comprising 11 channels, each with 22 resistors in series (462 resistors in total, Fig. S4). Apart from the edges, each network node has four resistive connections to neighbouring nodes (square configuration). Each resistor is randomly assigned one of two resistance values, *R*_A_ or *R*_V_ (*R*_V_ = 10^3^*R*_A_), with *p*_A_ representing the fraction of the total number of resistors that have been assigned a “low” resistance *R*_A_. For each network configuration, 50 simulation runs were performed, yielding a distribution of values for the network resistance *R*_NET_ and the corresponding conductance, *G*_NET_ = 1/*R*_NET_. Supervised machine learning was performed using a fine-grained K-nearest neighbor (KNN) classification model from Classification Learner toolbox in MATLAB.

## 3 Results and discussion

Fig. 1a, c and e depict the processes for α-Fe_2_O_3_ nanorod synthesis, LIG electrode fabrication, and device assembly, respectively, with corresponding SEM data shown in Fig. 1b, d and g. The Fe_2_O_3_ nanorods self-aggregate into a disordered network, as expected for polydisperse nanorods drop-deposited onto a polymer substrate (Fig. 1g). The nanorod morphology (Fig. 1b, inset) and the micropores formed through self-aggregation onto the polyimide surface between LIG electrodes (Fig. 1g) both increase the surface-to-volume ratio, providing more interaction sites for gaseous VOC molecules *vs.* thin films.^33–35^ SEM analysis of >50 nanorods yields an average length ∼215 ± 90 nm (Fig. S1a) and widths ∼50–150 nm.

**Fig. 2:**
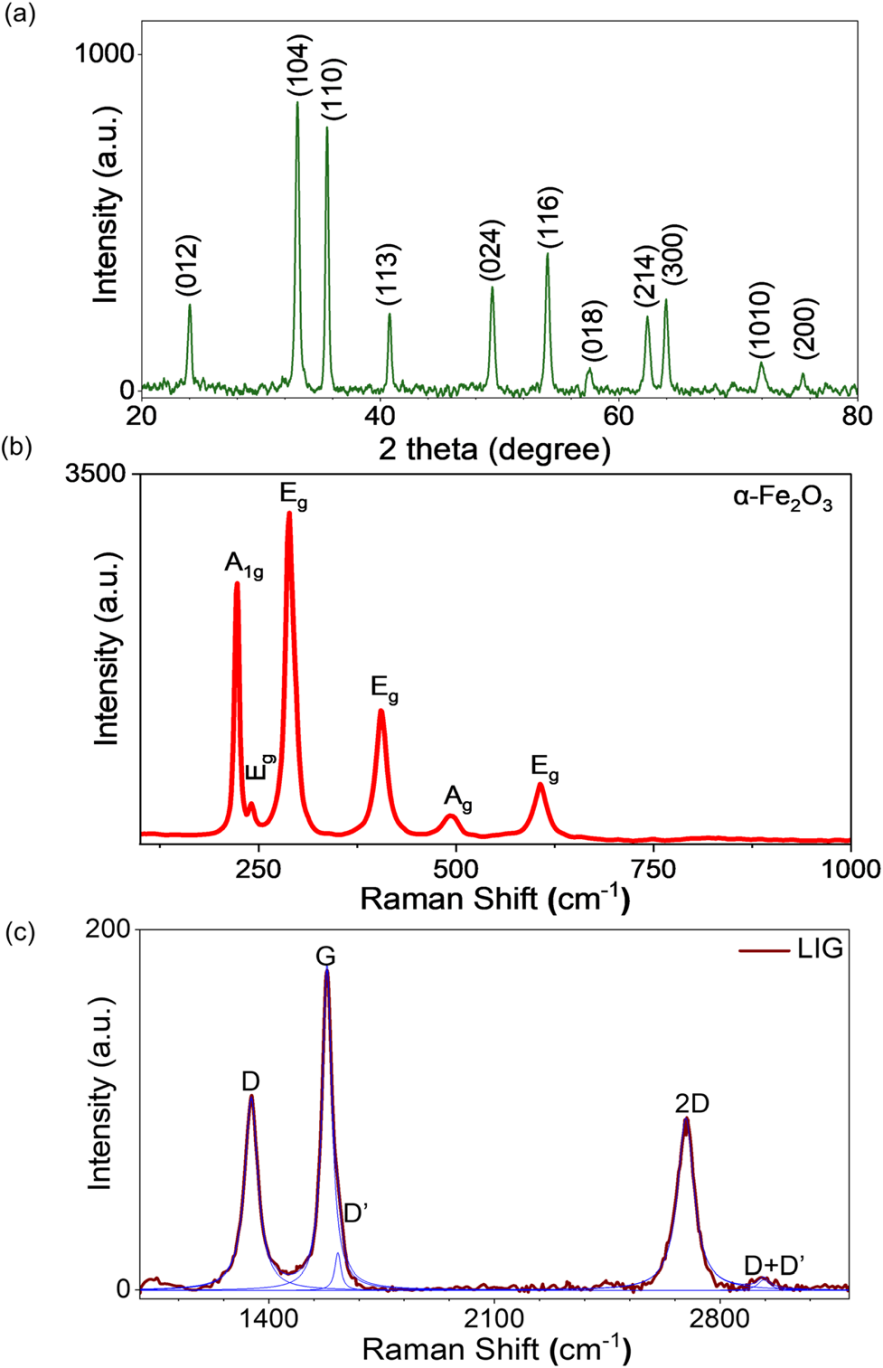
(a) Representative XRD for α-Fe_2_O_3_ nanorods. Representative Raman spectra for: (b) α-Fe_2_O_3_ nanorods, (c) LIG.^34,35^

The nanorods' phase and crystal structure were examined using X-ray diffraction (XRD) analysis. Fig. 2a shows the XRD 2*θ* peaks at 24°, 33°, 35°, 41°, 49°, 54°, 57°, 62°, and 64°, corresponding to (012), (104), (110), (113), (024), (116), (018), (214), (300), (1010), and (200) crystallographic planes, respectively. The data show good agreement with the typical trigonal crystal structure of hematite α-Fe_2_O_3_ (JCPDS card No: 33-0664) with space group *R*3̄*c*. No peaks related to other crystal phases or impurities were detected. Raman data (Fig. 2b) showed clear peaks for the expected modes for α-Fe_2_O_3_: *A*_1g_ (223 cm^−1^,495 cm^−1^) and *E*_g_(242 cm^−1^, 289 cm^−1^, 406 cm^−1^, 608 cm^−1^). No discernible peaks were observed for impurities or other iron oxide phases. EDX elemental analysis of individual α-Fe_2_O_3_ nanorods (Fig. S1b) again confirmed the presence of iron and oxygen with no other impurities detected.

A representative Raman spectrum from the LIG contact electrodes (Fig. 2c) shows the expected first-order peaks (D, D′, G) and the second-order 2D peak characteristic of multi-layer graphene-like carbon LIG.^31,36,37^ Table S2 summarizes the results extracted from Lorentzian fits to the data, confirming sharp peaks indicative of high-quality LIG, with full-width at half-maximum intensity (FWHM) values comparable to those previously reported using the same laser system: FWHM_D_ < 50 cm^−1^, FWHM_G_ < 40 cm^−1^, FWHM_2D_ < 70 cm^−1^.^31^

The conversion of polyimide film to graphene-like carbon is thought to involve both photothermal and photochemical processes, with the photothermal process likely playing a key role in breaking and reforming the bonds between carbon, oxygen, and nitrogen atoms at the polyimide surface.^38,39^ This process results in a color change of the orange polyimide tape to deep black, which is a good visual indication of carbonization/graphitization.^31^ The LIG electrode morphology (Fig. 1d and g) shows kinked and wrinkled regions exhibiting a hierarchical porous structure, ascribed to rapid generation of gaseous products during laser melting/vaporisation of polyimide and subsequent carbonization. EDX analysis of LIG (Fig. S1d) showed the expected strong carbon peak with trace amounts of oxygen and nitrogen.

### 3.1 VOC gas sensing using LIG-contacted α-Fe_2_O_3_ nanorods

#### 3.1.1 Influence of relative humidity

**Fig. 3:**
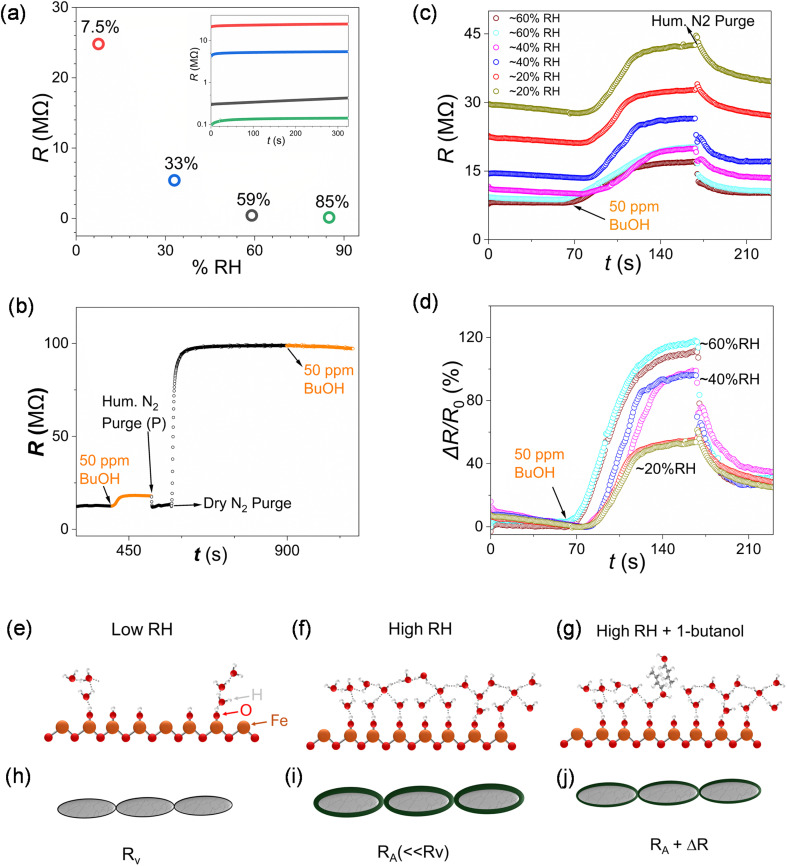
(a) Semi-log plot of measured saturated resistance *vs.* relative humidity for one LIG/α-Fe_2_O_3_ nanorod device (D17) in separate standard RH environments (saturated salt solutions). Inset: semi-log plot of resistance *vs.* time in each environment (b) resistance data for another device (D20) following injection of a 1 µL aliquot of 1-butanol (∼50 ppm) following by purging with humidified nitrogen (55 ± 5% RH) and dry nitrogen, respectively, and injection of a second 1 mL aliquot. (c) Simultaneously measured resistance data for two LIG-contacted α-Fe_2_O_3_ nanorod devices (D18, D19) towards 50 ppm of 1-butanol at 20 ± 5% RH, 40 ± 5% RH, and 60 ± 5% RH, respectively (*cf.* Fig. S6 and S12). (d) Corresponding Δ*R*/*R*_0_ data for D18 and D19. (e and f) Schematics of potential interactions of water molecules at α-Fe_2_O_3_ surfaces under low and high relative humidity (RH), respectively (g) Schematics of potential interactions of 1-butanol molecules at hydrated α-Fe_2_O_3_ surfaces. (h, i and j) Representation of nanorod assemblies considered as resistive cores (gray) with conductive shells (green) under the scenarios depicted in (e, f and g), respectively.

Understanding the influence of relative humidity on nanomaterial-based chemiresistive MOX VOC sensors operating at room temperature is of key importance. While charge transport in MOX sensors operating at high temperatures is often described in terms of processes mediated by oxygen radicals,^40^ Grotthuss-type protonic hopping transport across hydrogen-bonded networks of adsorbed water molecules is expected to play a significant role at room temperature.^41–44^ Studies of humidity sensing using nanostructured α-Fe_2_O_3_ thin films and chemically synthesised α-Fe_2_O_3_ nanomaterials have been widely reported over several decades. For this work, saturated salt solution standards were used to create environments with known relative humidity, specifically NaOH (7.5% RH), MgCl_2_ (33% RH), NaBr (59% RH), and KCl (85% RH). Fig. 3a shows the measured resistance for one LIG/α-Fe_2_O_3_ device (D17), acquired ∼5 minutes after insertion into each vessel. The saturation resistance shows the expected quasi-logarithmic dependence *vs.* relative humidity, decreasing by over two orders of magnitude from 7.5% RH to 85% RH. The ratio of the DC resistance at low and high humidity values, respectively, *R*_7.5%RH_/*R*_85%RH_ ∼210, is in reasonable agreement with data reported for assemblies of smaller α-Fe_2_O_3_ nanorods, *R*_11%RH_/*R*_92%RH_ ∼340.^45^Fig. 3e schematically depicts the established model for interaction of water molecules at hematite surfaces under low humidity. Hydroxylation of the surface occurs initially and arriving water molecules can then interact with surface –OH groups. Near-ambient X-ray photoelectron spectroscopy studies on single-crystal α-Fe_2_O_3_(0001) reported adsorption of the first complete water monolayer (ML) at ∼15% RH, with coverage increasing to 1.5 ML at 34% RH.^46^

Charge transport through α-Fe_2_O_3_ nanomaterials as a function of relative humidity can then be modelled by considering percolative conduction through a two-dimensional random resistor network.^47^ In this coarse-grained approach, each resistor corresponds to one mesoscopic domain of assembled nanorods (Fig. S4). In the simplest case, each domain can have one of two resistance values, *R*_A_ or *R*_V_. “Active” domains with a multi-layer surface coverage of adsorbed water, *i.e.* hydrogen-bonded networks that facilitate protonic transport are assigned a resistance value *R*_A_ (Fig. 3i). Domains with sparse, sub-monolayer coverage of adsorbed water, corresponding to vacancies or defects in the resistor network, are assigned a resistance value *R*_V_ (Fig. 3h), with *R*_V_ >> *R*_A_.

If there are *N*_A_ “active” domains and *N*_V_ “vacant” domains in a particular network configuration, the percolation fraction is defined as *p*_A_ = N_A_/(*N*_A_ + *N*_V_). For each simulation run, we randomly assign values of *R*_A_ or *R*_V_ to individual resistors to achieve the required percolation fraction, *p*_A_. The network resistance *R*_NET_ and the corresponding conductance, *G*_NET_ = 1/*R*_NET_, can then be calculated. Fig. S5a shows simulated, normalised conductance data, *G*/*G*_max_*vs. p*_A_, showing the expected “hockey-stick” shape with quasi-linear behavior at *p*_A_ values above the percolation threshold (*p*_A_ ∼0.4). Interestingly, normalized conductance data *vs.* relative humidity (Fig. S5b), extracted from the resistance data shown in Fig. 3a, show similar behavior. This suggests that charge transport through the LIG-contacted α-Fe_2_O_3_ devices at ambient humidity levels comprises multiple conducting paths mediated by a disordered network of hydrogen-bonded water molecules at the α-Fe_2_O_3_ nanorod surfaces (Fig. 3f and i). The dominant mechanism for prototropic charge migration through “freestanding” water networks features hydronium ions, H_3_O^+^ protonated water, that are triply hydrogen-bonded to neighbouring water molecules, *i.e.* H_3_O^+^(H_2_O)_3_.^48^ Recent neural-network-based molecular dynamics simulations reveal that proton transport in water is doubly gated by sequential hydrogen-bond exchange.^49^ The situation at porous oxide surfaces in the presence of electric fields is even more complex,^48^ with contributions from both H_3_O^+^ and OH^−^ ions. The measured device resistance also reflects combined effects of two distinct Grotthuss mechanisms: (i) vehicular diffusion, *i.e.*, ion migration; (ii) structural diffusion, *i.e.* charge migration *via* proton exchange, *e.g.* (A^+^)(B) → (A)(B^+^). Our results are consistent with these mechanisms, where humidity-driven increases in the water layer thickness at α-Fe_2_O_3_ nanorod surfaces lead to improvements in local co-ordination of the hydrogen-bonded network, thus improving charge migration and reducing device resistance. The influence of VOCs on prototropic charge transport through these hydrogen-bonded networks will be discussed below.

An affordable, custom-made gas-sensing setup was used to assess the chemiresistive behavior of the LIG-contacted α-Fe_2_O_3_ nanorod assemblies towards a range of VOCs under different humidity conditions (Fig. S2). Before conducting analyte tests, the test chamber was flushed with humidified nitrogen (20 ± 5% RH) in the presence of the device(s) for 5 min to stabilize the sensor devices and remove impurities. Fig. 3b shows a semi-log plot of the measured DC resistance (*R*) for one device (D20) to a 1 µL injection of 1-butanol (∼50 ppm vapor concentration, see Fig. S3a). From the initial resistance, *R*_0_ ∼12 MΩ, the device resistance increased following injection of the 1-butanol aliquot to a plateau value, ∼18 MΩ. Upon purging the chamber with humidified nitrogen (55 ± 5% RH), the resistance fell rapidly and stabilized at ∼13 MΩ, close to the initial value. Subsequent purging with dry nitrogen (<5% RH) resulted in a rapid, significant increase in resistance, to ∼99 MΩ, consistent with desorption of surface water molecules and a reduction in the number of viable charge transport paths through the α-Fe_2_O_3_ nanorod assembly. Injection of a 1-butanol aliquot did not lead to any significant change in device resistance. Similar behavior was observed for a second device (D21, Fig. S8a) measured simultaneously with D20.

#### 3.1.2 Sensitivity & reproducibility

Measurements for two devices (D18, D19) mounted together in the sensing chamber showed similar trends over three successive measurement runs at relative humidity values of 60 ± 5% RH, 40 ± 5% RH and 20 ± 5% RH, respectively (Fig. 3c). For both devices, the initial resistance (*R*_0_) increases with decreasing relative humidity (Fig. 3b inset, Fig. S6 and S7). Fig. 3d shows the resistance response Δ*R*/*R*_0_, *i.e.* the change in resistance as a percentage of the initial resistance for the data shown in Fig. 3c. The response to 50 ppm 1-butanol decreased at lower relative humidity for both devices, with Δ*R*/*R*_0_ ∼110–117% at 60 ± 5% RH, decreasing to ∼97–98% at 40 ± 5% RH and falling strongly to ∼53–55% at 20 ± 5% RH. Taken together, these data highlight the key role of humidity on device performance and highlights good sensitivity close to ambient humidity levels.

**Fig. 4:**
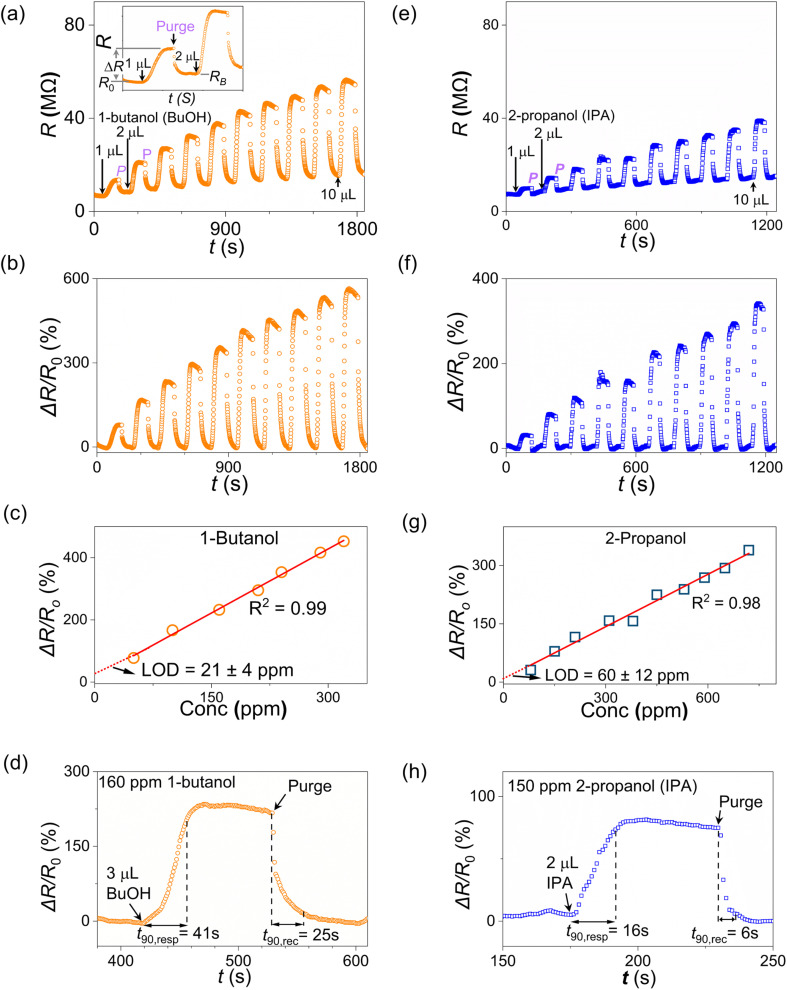
(a) Measured resistance data (*R*) *vs.* time (*t*) for one of four LIG-contacted α-Fe_2_O_3_ nanorod sensors (D2) simultaneously measured in a humidified nitrogen environment (∼55% RH) towards a series of injected aliquots of 1-butanol, from 1 µL to 10 mL; see Fig. S9 for full dataset (D1–D4). Inset: *R vs. t* data for first two measurement cycles, showing initial resistance (*R*_0_), change in resistance after injection of 1-butanol aliquot (Δ*R*) and drift of baseline resistance (*R*_0_ → *R*_B_) following purging with humidified nitrogen. (b) Δ*R*/*R*_0_ response calculated from resistance data after background subtraction, *cf.* Fig. S9b. (c) Peak response values *vs.* 1-butanol concentration for this device (D2) over linear dynamic range with linear fit used to extract the LOD. (d) Response data for device D2 showing *t*_90_ time constants for response to a 3 µL aliquot of 1-butanol (160 ppm); and recovery following purging with humidified nitrogen. (e and f) Resistance and Δ*R*/*R*_0_ response data for the same device towards increasing injection volumes of 2-propanol (isopropyl alcohol, IPA). (e) LOD fit from peak response *vs.* IPA concentration (f) response *vs.* time to a 2 µL aliquot (150 ppm) of IPA showing *t*_90_ time constants.

Two sets of measurements, each featuring four devices measured simultaneously (D1–D4, D5–D8), were undertaken on to systematically assess device performance and sensitivity to 1-butanol and other VOCs. Fig. 4a shows the measured DC resistance (*R*) for device D2 towards sequential injections of increasing volumes of 1-butanol, from 1 µL (∼50 ppm vapor concentration) to 10 µL (∼460 ppm), interspersed with humidified nitrogen purge cycles (55 ± 5% RH). From initial device resistance values in the range 7–9 MΩ, all four devices show significant resistance increases upon exposure to 1-butanol (Δ*R* in Fig. 4a inset, Fig. S9a). Following purging with humidified nitrogen, the device resistance decreased and settled at a baseline value *R*_B_. All devices showed a slight increase in baseline resistance (∼9–15%) after each injection-purge cycle. Control measurements on separate “blank” devices subjected to wait-purge cycles only, *i.e.* no analyte aliquots injected (Fig. S10a), showed similar increases in measured baseline resistance (∼8–10%). This baseline drift is consistent with cumulative surface dehydration due to the purge cycles (Fig. S10b).

The device with data shown in Fig. 4a (D2) showed a significant resistance response, Δ*R*/*R*_0_ ∼75% to 50 ppm 1-butanol. Measured response increased with concentration for all four devices within a linear dynamic range up to 300 ppm (Fig. 4b and S9c). For each device (D1–D4), the limit of detection (LOD) was calculated from a least-squares linear fit of measured response *vs.* VOC concentration (Fig. 3c) using eqn (1) as per the standard error estimate method,1
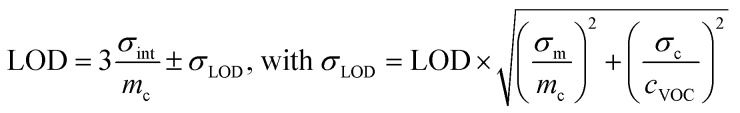
where *σ*_int_ is error on the intercept for the least-squares linear fit, *m*_c_ is the fit slope, *σ*_m_ is the error on the slope; and *σ*_c_/*c*_VOC_ is the fractional error in the VOC concentration (∼20% for these manual microsyringes). Table 2 shows the extracted 1-butanol LOD values for D1–D4, with calculated values in the range 21–33 ppm. Table S3a shows the full parameter set used for the calculations.

**Table 2:** (LOD) for each device calculated over the linear dynamic range (LDR): 50–300 ppm for 1-butanol, 80–500 ppm for IPA

Device	1-Butanol LOD (ppm)	2-Propanol LOD (ppm)
D1	32 ± 7	52 ± 11
D2	21 ± 4	60 ± 12
D3	29 ± 6	65 ± 13
D4	33 ± 7	54 ± 11
D5	49 ± 11	71 ± 15
D6	55 ± 12	43 ± 9
D7	29 ± 6	38 ± 8
D8	39 ± 8	67 ± 14

Fig. 4d also highlights the rapid, room-temperature response of the sensors towards the analyte for a typical cycle. The *t*_90_ response time is taken as the time for the Δ*R*/*R*_0_ resistance response to reach 90% of the maximum value for that cycle. Fig. S11a shows the extracted *t*_90_ response times *vs.* concentration for the four devices. The average response time across the four devices towards 160 ppm of 1-butanol is *t*_90,resp,BuOH_ (160 ppm) ≈ 40 ± 2 s. As expected, devices showed more rapid *t*_90_ recovery times after purging, with a mean value *t*_90,rec,BuOH_ (160 ppm) ≈ 25 ± 3 s.

After 24 hours under ambient conditions, these same devices were subsequently exposed to sequential injections of increasing volumes of 2-propanol (IPA), from 1 µL (∼80 ppm, see Fig. 4e and S3b) to 10 µL (∼720 ppm), interspersed with purge cycles. Again, all four devices (D1–D4) show significant resistance increases upon exposure to IPA (Fig. S9d). All devices show smaller Δ*R*/*R*_0_ response magnitudes to IPA *vs.* 1-butanol (Fig. 4f and S9e), *e.g.*, device D2 shows Δ*R*/*R*_0_ ∼31% to 80 ppm IPA. Device responses increased with increasing IPA concentration (Fig. S9f), with extracted LOD values in the range 52–65 ppm (Table S2a). The average response time across the four devices was lower for IPA *vs.* 1-butanol (Fig. S11b) with *t*_90,resp,IPA_ ≈ 20 ± 4 s for 150 ppm IPA. Devices showed even shorter *t*_90_ recovery times after purging, *t*_90,rec,IPA_ ≈ 8 ± 2 s.

A separate set of devices (D5–D8) was used to measure the responses to increasing concentrations of IPA first (Fig. S12a–c), and then 24 hours later to increasing concentrations of 1-butanol (Fig. S12d–f). These devices showed similar responses to the first set (D1–D4), with a slightly wider range of LOD values, 38–71 ppm for IPA (, see Table 2). These devices also showed a slightly wider range of LOD values for 1-butanol, 29–55 ppm. Response time constants (Fig. S11c and d) were comparable to the first set of devices: *t*_90,resp,BuOH_ (160 ppm) ≈ 34 ± 4 s and *t*_90,resp,IPA_ (150 ppm) ≈ 21 ± 3 s. While the response time is aliased by the different evaporation conditions for the various solvents, *e.g.*, solvent boiling point *vs.* hotplate temperature, the clear difference in *t*_90_ values suggests that the temporal response behavior could provide a potential route to discriminate between different solvents, as will be discussed below^50^

These resource-efficient, LIG-contacted α-Fe_2_O_3_ nanorod devices show excellent performance at room temperature, with mean response Δ*R*/*R*_0_ = 185 ± 25% to 100 ppm 1-butanol at ∼55% RH for D1–D8. The closest comparable literature report known to the authors, for room-temperature sensing of 100 ppm 1-butanol, Δ*R*/*R*_0_ ∼ −170% (30% RH) for a composite sensor, featuring α-Fe_2_O_3_ nanocubes combined with resource-intensive reduced graphene oxide (Table 1), with a response Δ*R*/*R*_0_ ∼ −13% reported for sensors featuring only the α-Fe_2_O_3_ nanocubes.^6^

**Fig. 5:**
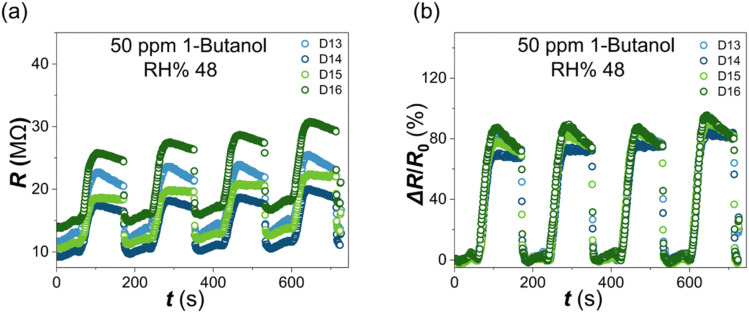
(a) Measured room-temperature resistance data *vs.* time for four α-Fe_2_O_3_ nanorod sensors (D13–D16) mounted together in the sensing chamber towards sequential injections of 1 µL 1-butanol (∼50 ppm), interspersed with humidified nitrogen purge cycles (b) corresponding normalised change in resistance (response), Δ*R*/*R*_0_, after baseline subtraction.

The LIG-contacted α-Fe_2_O_3_ nanorod devices also demonstrated reproducible behavior. Fig. 5a and b shows measured resistance and corresponding Δ*R*/*R*_0_ response data, respectively, for 4 devices (D13–D16) exposed to multiple injections of 1-butanol 1 µL, ∼50 ppm, Δ*R*/*R*_0_ ∼83 ± 1% for 16 injection cycles across 4 devices. The response data show low values for the Coefficient of Variation, CoV = *σ*/*µ*, where *σ* is the mean and *µ* is the standard deviation: 0.05 < CoV < 0.1 for device-to-device variation; and 0.05 < CoV < 0.08 for cycle-to-cycle variation. Our LIG/α-Fe_2_O_3_ devices also showed good linearity with linear dynamic range (LDR) from 50–300 ppm for 1-butanol and 80–500 ppm for IPA. Extracted LOD values for 1-butanol were in the range 21–55 ppm across the 8 devices (Table 2), all below the NIOSH 8-hour workplace exposure limit (100 ppm).

Two other important parameters were also considered: carrier gas (nitrogen *vs.* air) and α-Fe_2_O_3_ nanorod calcination temperature. A set of four devices (D24–D27) was first exposed to a series of injected 1-butanol aliquots (1 µL, 2 µL, 5 µL) in a humidified nitrogen environment (∼60% RH) with humidified nitrogen purging (Fig. S13a–c), followed by exposure to a second injection series in a humidified air environment with humidified air purging (∼60% RH, Fig S13d–f). Comparable device performance was observed between the two environments, highlighting the dominant role of relative humidity over carrier gas. Across the four devices, the percentage drift in baseline resistance arising from repeated purge cycles was comparable for measurements in humidified N_2_*vs.* humidified air, ∼60–110% in both cases. Similarly, comparable VOC response values were obtained for both environments: Δ*R*/*R*_0_ ∼240 ± 11% to 160 ppm 1-butanol in humidified N_2_*vs.* 244 ± 12% for the same devices in humidified air. Response time constants also showed good agreement, with *t*_90_ values ranging from 29–46 s in humidified N_2_ and *t*_90_ ∼29–58 s in humidified air (Fig. S13g and h). Finally, mean LOD values were also consistent across both carrier gas environments (Table S3b): LOD = 36 ± 14 ppm for 1-butanol under humidified nitrogen *vs.* 34 ± 14 ppm under humidified air. These data are also in reasonable agreement with other devices measured in humidified nitrogen, LOD_D1–D4_ = 29 ± 5 ppm and LOD_D5–D8_ = 43 ± 11 ppm.

Considering the influence of calcination temperature, *T*_calc_, devices fabricated from nanorod batches calcined at lower temperatures *T*_calc_ = 400 °C showed high baseline resistance, *R*_0_ ∼57 MΩ. Baseline resistance values decreased with increasing *T*_calc_, falling sharply to *R*_0_ ∼10 MΩ for *T*_calc_ = 550 °C with a further gradual reduction to *R*_0_ ∼8 MΩ for *T*_calc_ = 650 °C (Fig. S14a and b). Within the humidity-assisted percolation picture developed above, higher *T*_calc_ could enhance crystallinity and reduce the density of scattering centres,^51^ thus lowering *R*_0_ by creating additional percolation paths through the hydrogen-bonded network at hydrated α-Fe_2_O_3_ surfaces across the nanorod assembly. For VOC sensing, increasing *T*_calc_ could also increase the number of suitable molecular interaction sites at the α-Fe_2_O_3_ surfaces. If arriving VOC molecules created additional scattering centres at sites along conducting paths, this would lead to an increase in Δ*R* and therefore Δ*R*/*R*_0_. While measured response values for 1-butanol increased monotonically with *T*_calc_, the largest jump occurred between *T*_calc_ = 550 °C and *T*_calc_ = 600 °C (Fig. S14c and d). We therefore selected *T*_calc_ = 600 °C as the synthesis condition of choice: It delivers near-maximal response, Δ*R*/*R*_0_ ∼210% to 100 ppm 1-butanol, with a reduced thermal budget *versus T*_calc_ = 650 °C, thus optimizing device performance *vs.* cumulative energy demand.

#### 3.1.3 Selectivity

Three devices (D22–D24) were simultaneously exposed to a series of VOCs to investigate the selectivity of our LIG-contacted a-Fe_2_O_3_ nanorod devices. Seven VOCs were studied using a polar/non-polar sequence: methanol, acetone, ethanol, hexane, IPA, toluene and 1-butanol. For each VOC, two aliquots were injected, 1 mL and 3 mL, respectively, separated by a purge cycle with humidified nitrogen (*P*). Fig. 5a shows the baseline-subtracted resistance response Δ*R*/*R*_0_*vs.* time for D22; see Fig. S15 for resistance and response data for all 3 devices. All alcohol-VOCs showed strong Δ*R*/*R*_0_ responses (Fig. S15), while no appreciable response was observed for the non-polar VOCs: acetone, hexane and toluene.

**Fig. 6:**
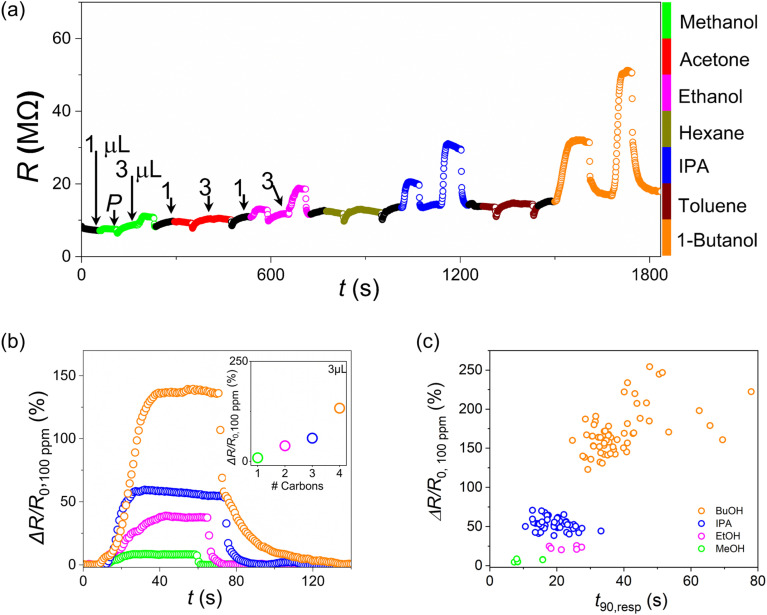
(a) Resistance response data, Δ*R*/*R*_0_ for one device (D22) to a sequence of polar and non-polar VOCs; comprising 1 mL injection, purging with humidified nitrogen (*P*, ∼60% RH) and 3 µL injection for each VOC. (b) Response per 100 ppm of analyte, Δ*R*/*R*_0,100 ppm_ for device D23 to injection of 3 µL aliquots for the alcohol VOCs, plotted from the start of each injection cycle. Inset: mean saturation value for Δ*R*/*R*_0,100 ppm_ for D22–D24 *vs.* no. of carbons, *i.e.* methanol (1) to 1-butanol (4); see Table S4. (c) Concentration-normalized resistance response, Δ*R*/*R*_0,100 ppm_*vs. t*_90_ response time for D1–D8, D9–D12, D22–D24.

In order to compare the responses for the different alcohols, we consider the concentration-normalized response for each VOC, Δ*R*/*R*_0,100 ppm_, defined as the resistance response per 100 ppm of analyte, (Table S4 and Fig. 6b). This concentration-normalized response shows a non-linear dependence on the number of carbons (Fig. 6b inset), with a significantly stronger response for 1-butanol. For each VOC, all three devices show similar concentration-normalized responses for the 3 mL aliquots with coefficients of variation, CoV < 0.1 for 1-butanol, IPA and ethanol; and CoV < 0.15 for methanol. The mean Δ*R*/*R*_0@100 ppm_ values across the three devices for 1-butanol (143 ± 11%) and IPA (62 ± 4%) are in reasonable agreement with corresponding values extracted from the slope of the response *vs.* concentration curve, *m*_c_, for devices D1–D8 (Table S3a): Taking an estimate of Δ*R*/*R*_0,100 ppm_ ≈ 100 *m*_c_ yields values in the range 109–138% for 1-butanol and 39–48% for 2-propanol. These LIG-contacted a-Fe_2_O_3_ nanorod devices also show good resistance response selectivity when compared to other chemiresistive sensors targeting detection of 1-butanol, see Table S5.

Current–voltage (*I*–*V*) measurements were acquired for a device exposed to a series of high vapor concentrations (Fig. S16). The first measurement in humidified air (KCl standard, 85% RH) showed the expected hysteretic behavior for a high humidity environment with ∼6 µA current measured at 5 V. Subsequent measurements in different VOC environments IPA, acetone, ethanol (EtOH), 1-butanol (BuOH) showed lower hysteresis and a trend in measured currents that matched the low-bias resistance data shown in Fig. S16a with *I*_85%RH_ > *I*_acetone_ > *I*_EtOH_ > *I*_IPA_ > *I*_BuOH_ at 5 V. This supports our assertion that interaction of the alcohol VOCs with the hydrated a-Fe_2_O_3_ surfaces impedes charge transport through the nanorod assembly (Fig. 3j), likely *via* reducing the net carrier concentration and/or carrier mobility. Further work is needed to elucidate the relative contributions of these mechanisms.

In addition to the magnitude of the concentration-normalized response, we also observe different time signatures for each VOC. Fig. 6b shows the concentration-normalized device response, Δ*R*/*R*_0,100 ppm_, *vs.* time elapsed after VOC injection, *t*–*t*_0_, for D23. Following injection of 1-butanol, Δ*R*/*R*_0, 100 ppm_ continues to increase to a significantly higher magnitude and over a longer period of time compared to the response for the same device to 2-propanol, ethanol or methanol. Similarly, the time constants for recovery after purging, *t*_90,rec_, are significantly larger for 1-butanol *vs.* the other VOCs. Fig. 6c shows Δ*R*/*R*_0,100 ppm_*vs.* the response time constant following VOC injection, *t*_90,resp_, for a range of devices and VOCs. The data show clear evidence of clustering for the 1-butanol and IPA data. It is interesting to note that while the outliers for the 1-butanol cluster, *t*_90,resp_ ≥ 48 s, are all from the first 1-butanol scans (50 ppm) for D1–D8 (Table S6), the same trend is not observed in the first IPA scans for the same devices (Table S7), apart from the first IPA scan for D1 (*t*_90,resp_ = 33 s). Fig. S17 shows fine-grained K-nearest neighbor (KNN) classification model results for the data shown in Fig. 6c using the concentration-normalized response, Δ*R*/*R*_0,100 ppm_, and the *t*_90_ time constants for response to VOC injection and recovery after purging, *t*_90,resp_ and *t*_90,rec_, respectively (Tables S6–S9). The model shows clear discrimination between the datasets for 1-butanol (*n* = 62) and IPA (*n* = 46). Given the small dataset size for ethanol and methanol (both *n* = 6), more data is needed to assess the selectivity between the shorter-chain alcohols rigorously. Similar machine-learning-based approaches, such as KNN and PCA-assisted classification, have been successfully employed to distinguish multiple gas species and concentrations in mixed environments using single chemiresistive sensors.^52,53^

#### 3.1.4 VOC sensing mechanism and influence of relative humidity

While machine learning approaches can generate “black box” models linking VOC-dependent device response magnitude and time constants, it is useful to consider surface molecular interaction mechanisms that could influence device performance. Experimental and modelling studies of adsorption of short-chain alcohols at aqueous solution–air interfaces, *e.g.*, aerosols,^54,55^ reveal an increase in the Gibbs free energy for adsorption, Δ*G*_ads_^0^, as the alkyl chain length increases. Reported room-temperature (290 K) values range from Δ*G*_ads_^0^ = −6.5 kJ mol^−1^ (∼70 meV per molecule) for methanol to Δ*G*_ads_^0^ −15.3 kJ mol^−1^ (∼170 meV) for 1-butanol, compared to the thermal energy *k*_B_*T* ≈ 25 meV at room temperature. If thermally-activated processes dominated the molecular residence time at the surface, *t*_res_, this would result in an almost 11-fold increase in residence time for 1-butanol molecules *vs.* methanol molecules.

Ultrafast THz spectroscopy studies have provided insight into the hydrogen-bond structure and dynamics in alcohol-water mixtures for both fully-soluble alcohols – methanol, ethanol, 2-propanol (IPA) – and partially-soluble alcohols, 1-butanol.^56,57^ Moving from methanol to 1-butanol, *i.e.*, increasing hydrophobicity, recent THz time-domain spectroscopy (TTDS) data show that preferential hydrophobic chain–chain interactions lead to formation of 1-butanol aggregates in alcohol-water binary mixtures. Such aggregates could increase device resistance by increasing the effective path length for charge migration at the hydrated α-Fe_2_O_3_ nanorod surface. This scenario suggests that both the hydrophilic –OH head-group interaction with the hydrated α-Fe_2_O_3_ surface and the hydrophobic alkyl chain–chain intermolecular interactions contribute to the VOC interaction energy (and thus molecule residence time) since no appreciable resistance changes were observed for devices exposed to 1-hexane, a non-polar chain alkane that is insoluble in water (Fig. 6a and S15a). The reported TTDS data complement previous THz-calorimetry results, which suggest that increasing alcohol chain length (methanol to butanol) shifts hydration water from more tetrahedral toward more interstitial/defective configurations.^56^ This increased disorder would reduce the number of viable charge migration paths, thus increasing the chemiresistive response, Δ*R*/*R*_0_. The time evolution of the response for different VOCs (Fig. 6b and c) also supports this picture, with the larger response magnitude and increased *t*_90_ time constants consistent with gradual aggregation of 1-butanol molecules around initial nucleation sites.

We therefore attribute the chemiresistive response to reduction in the number of prototropic charge migration paths at the hydrated nanorod surfaces (Fig. 3g). Mechanistically, higher relative humidity lowers *R*_0_ by activating more water-bridged paths (network above percolation threshold) and increases Δ*R*/*R*_0_ because alcohol molecules arriving at the device surface can perturb a larger fraction of those viable paths. At low relative humidity, the hydrogen-bonded interfacial water network is below the percolation threshold so arriving alcohol molecules which interact with already “broken” paths won't cause any further increase in device resistance. Jo *et al.* likewise reported similar behaviour in MOF-based chemiresistive sensors, with electronic charge transport dominating at low relative humidity below the percolation threshold (∼25% RH) and prototropic conductivity dominating at high RH.^58^

Considering future practical applications, the contribution of relative humidity to chemiresistive VOC sensor device performance and sensitivity is often significant at room temperature.^58–60^ Therefore, field-deployable chemiresistive sensing systems would require pre-calibration/training in known humidity environments, which is common practice for commercial chemiresistive VOC sensors.^61^ Such systems also require a “humidity-only” sensor for simultaneous RH measurements in order to de-embed the contribution the relative humidity to the baseline resistance *R*_0_ (Fig. 3a) and the chemiresistive response Δ*R* (Fig. 3c).

Similar to commercial multi-device sensor array platforms,^62^ we expect that future, resource-efficient VOC sensor systems will feature multiple sensors with quasi-orthogonal response magnitude and time constants for target VOCs, together with standalone humidity and temperature sensors, in order to accurately discriminate target VOCs in real-world gas environments.

#### 3.1.6 Environmental footprint impacts

Finally, we return to the environmental footprint impacts of our LIG-contacted α-Fe_2_O_3_ nanorod sensors. We have demonstrated that our LIG/a-Fe_2_O_3_ hybrid sensors show good “3S” performance in terms of Sensitivity (Fig. 3, S9 and S12), Selectivity (Fig. 6 and S15) and Stability (Fig. 5). These devices also show clear potential for good performance under the important 4th S: Sustainability. In terms of comparative, order-of-magnitude assessment of key hotspots during the “Cradle to Gate” lifecycle phase (raw materials, processing and device fabrication, *cf.*Table 1), for the active material, reported Cumulative Energy Demand (CED) values for Fe_2_O_3_ from simple co-precipitation lie in the ∼20–200 MJ kg^−1^ range. For the contact electrodes, conventional Au electrodes account for ∼200 000 MJ kg^−1^ embodied energy for the source metal. Metal vacuum deposition adds a non-trivial per-coupon electricity burden (∼4.5 MJ here, scaled from a lab sputter dataset). By replacing Au/PVD with *in situ* LIG patterning, the per-coupon electricity is ∼0.01 MJ (measured facility draw for a 3 cm^2^ pattern), yielding orders-of-magnitude savings at the electrode level. Substrate choices are similarly important for CED: alumina/ceramic (∼80–1800 MJ kg^−1^, depending on route) *versus* glass (∼40 MJ kg^−1^) or polyimide (∼170–195 MJ kg^−1^). Thus, our LIG-contacted α-Fe_2_O_3_ nanorod devices on glass show significantly lower CED values *vs.* conventional chemiresistive MOX sensors. Further reductions in CED will focus on replacing the synthetic polyimide LIG feedstock and the glass substrate with abundant biopolymer substrates suitable for laser graphitization, *e.g.*, chitosan.^63^ We also note that room-temperature operation for our LIG/α-Fe_2_O_3_ devices will also reduce power consumption and thus CED during operation in the “Gate to Grave” lifecycle phase and improve prospects for short-lifetime sensor components, *e.g.*, for breath sensing or wearable health applications.

## 4 Conclusion

We have developed a low environmental footprint route for room-temperature chemiresistive detection of VOCs by combining α-Fe_2_O_3_ nanostructures derived from abundant raw materials and laser-induced graphene (LIG) contact electrodes. Initial order-of-magnitude comparative screening estimates indicate that these materials and processes will have significantly lower environmental impacts than traditional approaches. The α-Fe_2_O_3_/LIG sensors detect 1-butanol at occupational short term exposure limits (STEL, 50 ppm), with LOD ∼36 ± 11 ppm and mean Δ*R*/*R*_0_ ∼100 ± % (*n* = 8) at 50 ppm, and show a linear range of 50–300 ppm. Given the large Δ*R*/*R*_0_ values at 50 ppm, we expect that these sensors could address sub-10 ppm levels needed for domestic monitoring, with further scope for optimisation of nanorod surface area and contact electrode critical dimensions. The α-Fe_2_O_3_/LIG sensors also demonstrated rapid response times (*t*_90_ ≈ 40 ± 5 seconds) and recovery times (*t*_90_ ≈ 25 ± 3 seconds) for 160 ppm 1-butanol. Sensor response improved with increasing relative humidity (RH) across the ambient humidity range (20–60% RH), highlighting the key role of hydrogen-bonded networks of water molecules at α-Fe_2_O_3_ nanorod surfaces. Further, using resistance response magnitude and *t*_90_ time constants (response, recovery) yielded clear selectivity for 1-butanol *vs.* other VOCs. This sensor design combines rapid and sensitive detection with an environmentally-friendly fabrication process, showing excellent performance at room temperature. These sensors show good potential for affordable, sustainable VOC detection in growing application fields, including environmental monitoring and workplace safety.

## Author contributions

Mintesinot Tamiru Mengistu: methodology, investigation, data curation, visualization, and writing of the original draft. Richard Murray: supervision, methodology, review & editing, validation formal analysis. Alida Russo: formal analysis. Cathal Larrigy: methodology. Daniela Iacopino: supervision, review & editing. Michael Nolan: validation formal analysis. Colin Fitzpatrick: validation formal analysis. Aidan J. Quinn: supervision, resources, conceptualization, funding acquisition, analysis, MATLAB simulations, review & editing.

## Conflicts of interest

The authors declare that they have no financial interests or personal connections that may impact the results of this study.

## Supplementary Material

NA-008-D5NA00609K-s001

## Data Availability

The data supporting this article have been included as part of the supplementary information (SI). Supplementary information: Fig. S1–S18, Tables S1–S10 comprises: benchmarking of resistive response for LIG-contacted α-Fe_2_O_3_ nanorod devices towards 1-butanol against literature; SEM/EDX of α-Fe_2_O_3_ nanorods and LIG; Raman peak fit results for LIG; VOC sensing setup; VOC concentration measurements following aliquot injection; random-resistor-network simulations; simulated normalised conductance *vs.* percentage of active resistors; normalised device conductance *vs.* relative humidity (D17); baseline resistance and resistance change *vs.* relative humidity; influence of carrier gas humidity on baseline resistance and response to 1-butanol; VOC sensor performance (D1–D4); determination of limit of detection (D1–D8); effect of repeated purging using humidified nitrogen; sensor *t*_90_ response time constants following analyte injection; VOC sensor performance (D5–D8); VOC sensing in humidified nitrogen *vs.* humidified air; influence of calcination temperature, *T*_calc_, on baseline resistance and response to 1-butanol; selectivity of LIG-contacted α-Fe_2_O_3_ nanorod devices; concentration-normalised response for each VOC; comparison of response selectivity *vs.* literature; *I*–*V* measurements in different VOC environments; machine learning models for concentration-normalised resistance response and *t*_90_ times; device summary (D1–D33); UV-Vis absorption spectrum and Tauc plot for α-Fe_2_O_3_ nanorod solution. See DOI: https://doi.org/10.1039/d5na00609k.
