# Supplementary material for: Hematite α-Fe2O3 nanorods and laser-induced graphene for sustainable chemiresistive sensing of 1-butanol at room temperature
Source: Nanoscale Adv. 2025 Nov 5;8(1):192–206. doi: 10.1039/d5na00609k (PMC12645285; doi:10.1039/d5na00609k)
Supplement: NA-008-D5NA00609K-s001 [file NA-008-D5NA00609K-s001.pdf]

Supplementary Information (SI) for Nanoscale Advances.
This journal is © The Royal Society of Chemistry 2025

Supplementary Information

Nanoscale Advances

ROYAL SOCIETY OF CHEMISTRY

PAPER

Cite this: DOI: 10.1039/d5na00609k

Hematite α-Fe_2O_3 nanorods and laser-induced graphene for sustainable chemiresistive sensing of 1-butanol at room temperature

Mintesinot Tamiru Mengistu, [a] Richard Murray, [a] Alida Russo, [a] Cathal Larrigy, [a] Daniela Iacopino, [a] Colin Fitzpatrick, [b] Michael Nolan [a] and Aidan J. Quinn. [*a]

*Corresponding Author: aidan.quinn@tyndall.ie

[a]Tyndall National Institute, University College Cork, Lee Maltings, Dyke Parade, Cork T12 R5CP, Ireland.

[b] Dept of Electronic & Computer Engineering, University of Limerick, Limerick V94 T9PX, Ireland.

DOI: 10.1039/D5NA00609K

Table of Contents

Content	Figure/Table	Page(s)
Benchmarking of resistive response for LIG-contacted α-Fe_2O_3 nanorod devices towards 1-butanol against literature.	Table S1	3
SEM/EDX of α-Fe_2O_3 nanorods and LIG.	Figure S1	4
Raman peak fit results for LIG.	Table S2	5
VOC sensing setup.	Figure S2	5
VOC concentration measurements following aliquot injection.	Figure S3	6
Random-resistor-network simulations.	Figure S4	7
Simulated normalised conductance vs percentage of active resistors.	Figure S5a	7
Normalised device conductance vs relative humidity (D17).	Figure S5b	7
Baseline resistance and resistance change vs relative humidity.	Figure S6 (D18)<br>Figure S7 (D19)	8<br>9
Influence of carrier gas humidity on baseline resistance and response to 1-butanol.	Figure S8 (D21)	10
VOC sensor performance (D1–D4).	Figure S9	11
Determination of limit of detection (D1–D8).	Table S3	12–13
Effect of repeated purging using humidified nitrogen.	Figure S10	14
Sensor t_{90} response time constants following analyte injection.	Figure S11 (D1–D8)	15
VOC sensor performance (D5-D8).	Figure S12	16
VOC sensing in humidified nitrogen vs humidified air.	Figure S13	17–18
Influence of calcination temperature, T_{calc}, on baseline resistance and response to 1-butanol.	Figure S14 (D28–D33)	19
Selectivity of LIG-contacted α-Fe2O_3 nanorod devices.	Figure S15	20
Concentration-normalised response for each VOC.	Table S4	21
Comparison of response selectivity vs literature.	Table S5	22
I-V measurements in different VOC environments.	Figure S16	23
Machine Learning Models for Concentration-normalised Resistance Response and *t*90 times.	Figure S17<br>Table S6 (BuOH)<br>Table S7 (IPA)<br>Table S8 (EtOH)<br>Table S9 (MeOH)	24<br>25<br>27<br>28<br>28
Device summary (D1–D33).	Table S10	29
UV–Vis absorption spectrum and Tauc plot for α-Fe_2O_3 nanorod solution.	Figure S18	30
SI References.		31

Table S1. Comparison between 1-butanol responses of the present sensor and those reported in the literature.

Sensing material	Electrode	Substrate	Conc. (ppm)	Response R_{air}/R_{VOC}	Response $(R_{VOC}-R_0)/R_0$ (%)[a]	Temp. (°C)	RH (%)	Ref.
leaf-like α-Fe_2O_3	Au	Ceramic	100	~8		260	32	1
β-FeOOH/α-Fe_2O_3	Au	Ceramic	100	~1		250	-	2
AuNPs-modificated Fe_2O_3/$ZnFe_2O_4$	-	Ceramic	20	~18		160	60	3
MOF-derived Fe_2O_3	Au	Alumina	100	~10		230	30	4
ZnO decorated α-Fe_2O_3 nanorods	Au	Ceramic	100	~54		225	50-65	5
α-Fe_2O_3 Polyhedral nanoparticles	Au	Ceramic	100	~25		300	-	6
Bi_2WO_6/α-Fe_2O_3	Ag, Pd	Ceramic	50	~23		260	25	7
NiO/Fe_2O_3	Cr, Au	Alumina	10	~5		200	45	8
Al/$ZnFe_2O_4$	Au	Alumina	100	~19		175	40	9
MOF-nanocube Fe_2O_3	Au	Ceramic	100	-	~-12%	RT	30	10
MOF-nanocube Fe_2O_3/reduced Graphene Oxide (rGO)	Au	Ceramic	100	-	~-170%	RT	30	10
α-Fe_2O_3 nanorods	LIG	Polyimide	100	-	185 ± 25 %, n=8	RT ~20 °C	55-60	(this work)

[a] R_0 : Device resistance under ambient conditions (air or humidified nitrogen); R_{VOC} : Device resistance following exposeure to volatile organic compound (*VOC*) vapor.

Scanning electron microscopy (SEM) and Energy-Dispersive X-ray Spectroscopy (EDX) data

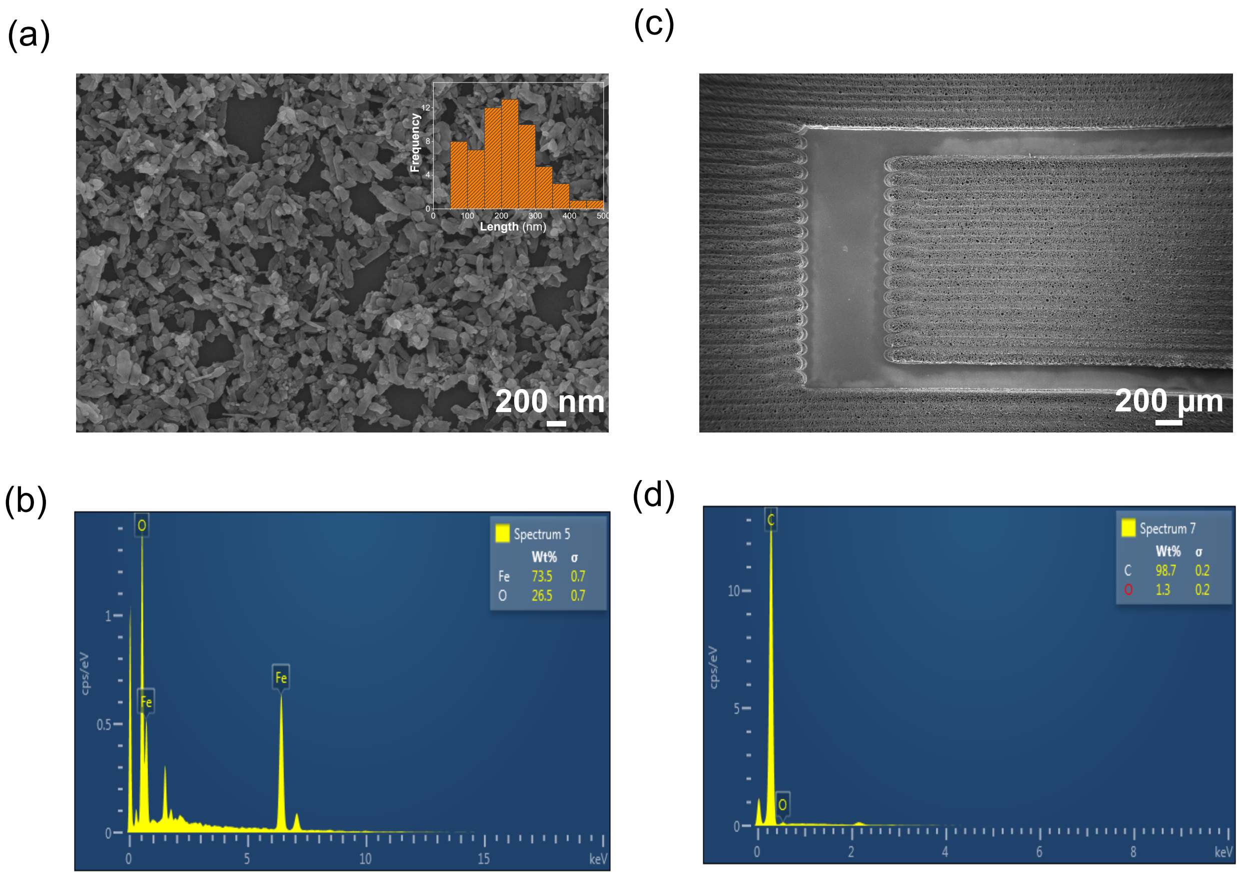

Figure S1. (a) SEM of drop-sposited α-Fe_2O_3 nanorods on Si(100) substrate inset: Nanorod length distribution. (b) Representative EDX spectrum of α-Fe_2O_3 nanorods. (c) SEM microstructure of LIG electrode (d) Representative EDX spectrum for LIG electrode.

Raman peak fit data

Table S2. Lorentzian fit characteristics to Raman data for *LIG* (Figure 2c). A_x/A_G refers to the peak area (x = *D*, *D'*, *2D*, *D'+D*) as a fraction of the area under the *G* peak.

**Raman peak**	**Peak position (cm^{-1})**	***FWHM* (cm^{-1})**	A_x/A_G
D	1346	50	0.80
G	1580	37	–
D'	1614	23	0.07
2D	2693	61	0.89
D +D'	2935	44	0.04

VOC sensing setup

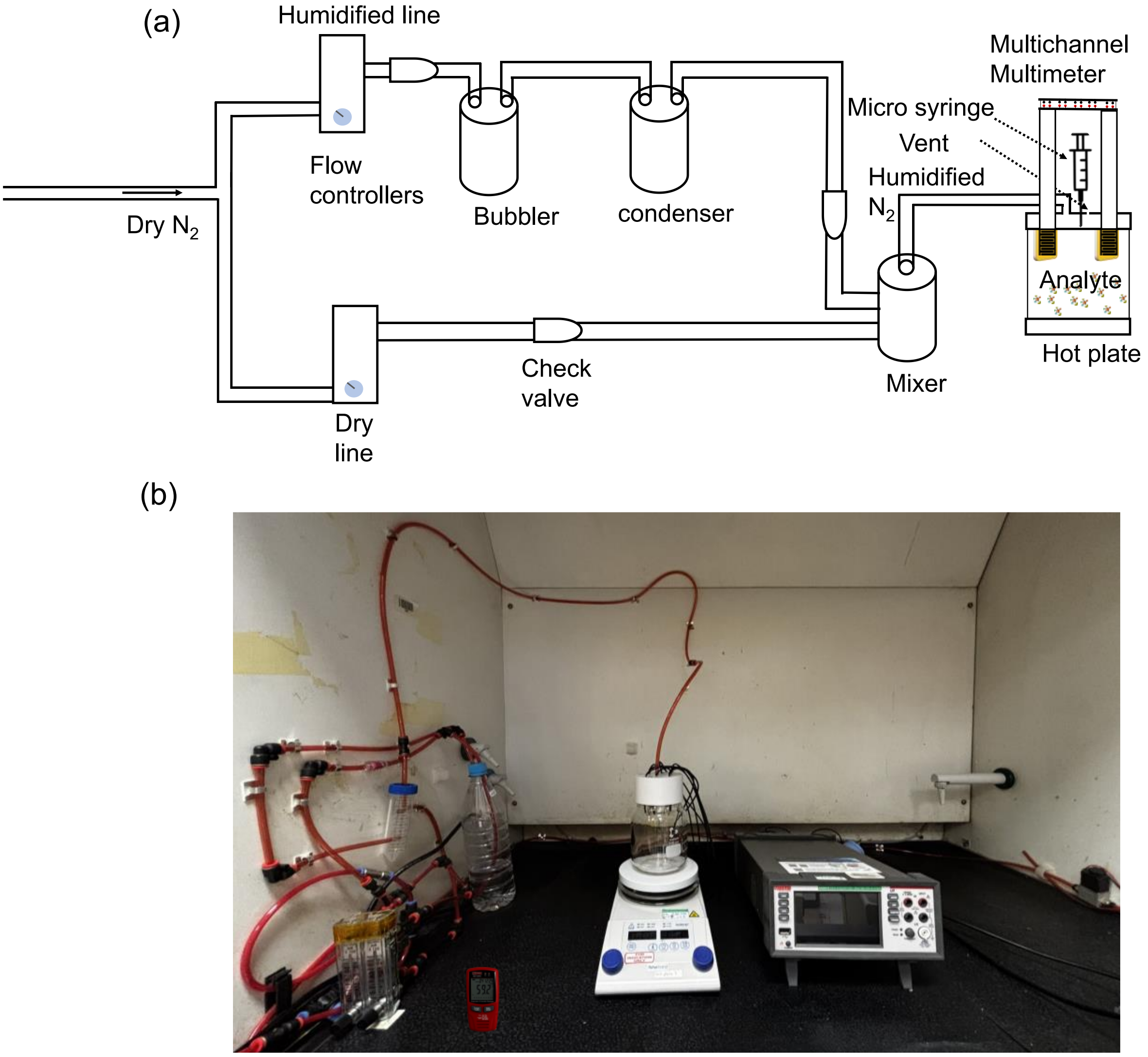

Figure S2. (a)Schematic of VOC sensing Setup (b) Photograph of VOC sensing setup.

VOC concentration measurements following aliquot injection

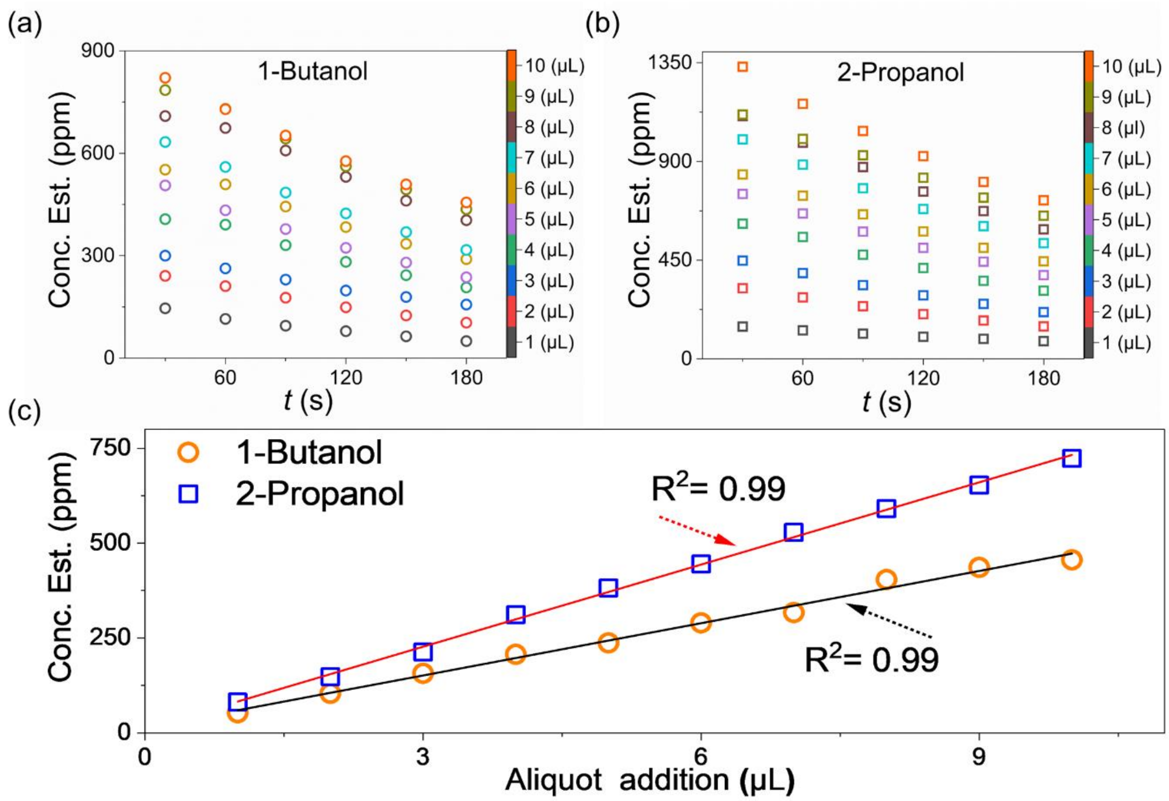

Figure S3. Time evolution of VOC concentration in the sensing chamber after injection of liquid analyte: (a) 1-butanol; (b) 2-propanol. (c) Linear fit of the 1-butanol and 2-propanol final concentrations (in ppm) measured 3 minutes after analyte injection.

Simulations of random resistor networks

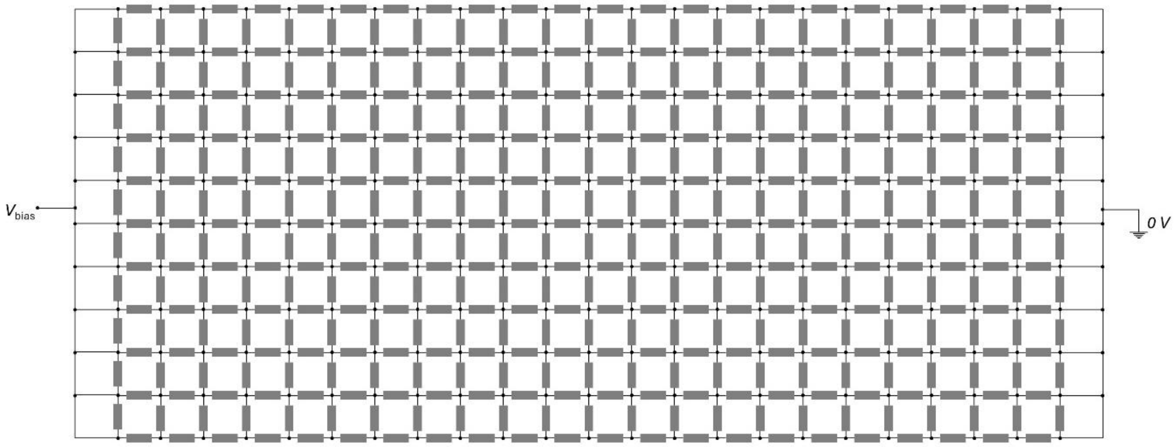

Figure S4. Resistor network used for MATLAB Simulink simulations, comprising 11 parallel channels, each with 22 resistors (462 resistors in total). Apart from the edges each network node has four resistive connections to neighbouring nodes (square configuration).

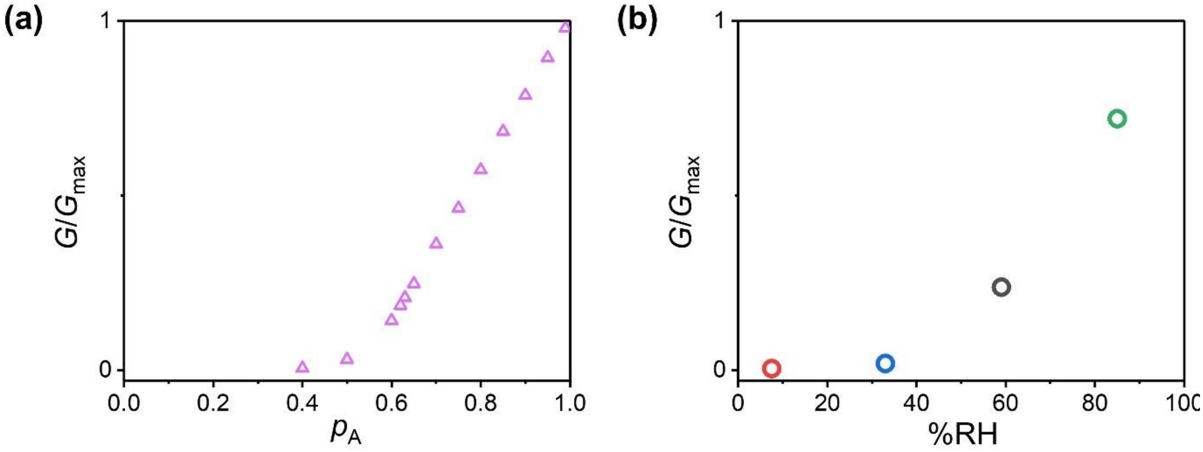

Figure S5. (a) Normalised conductance data simulated for 11×22 random resistor network. Each resistor is randomly assigned one of two values (R_A, R_V, with R_V =1000 R_A) and the total network conductance G is calculated vs the fraction of the resistors with resistance R_A, p_A. Each data point (triangles) is the average of 50 simulation runs. Maximum conductance G_{max} = $1/(2R_A)$ occurs at p_A = 1 (no disorder). (b) Normalised device conductance vs relative humidity calculated from measured resistance data for D17 (Fig 3a).

Baseline resistance and resistance change vs relative humidity

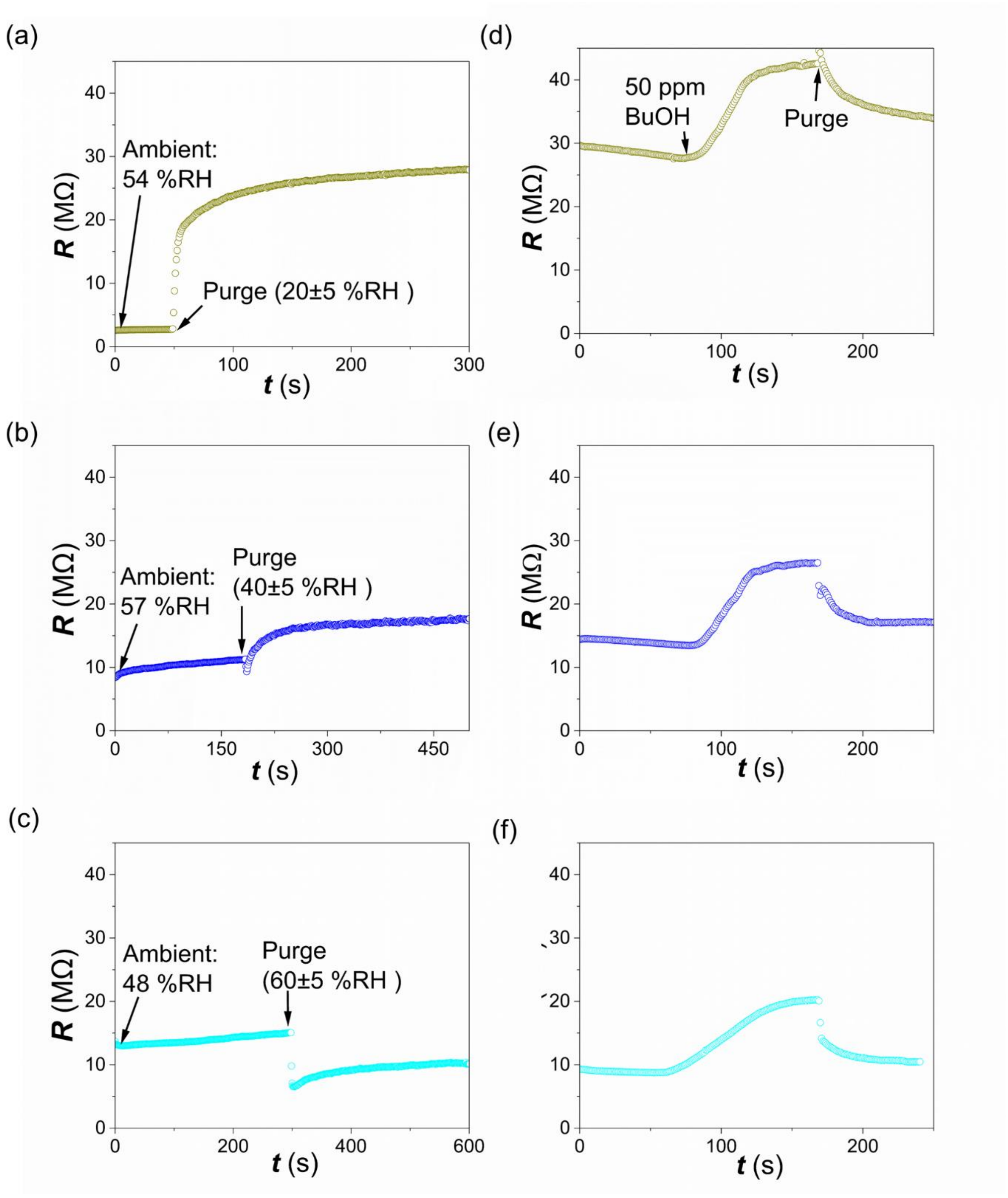

Figure S6. stabilization of the sensor through purging the humidified nitrogen to device mounted (D18) in the testing chamber to mimic desired humidity (a) 20% RH (b) 40% RH (c) 60% RH: Measured room-temperature resistance data vs time for α-Fe_2O_3 nanorod sensors mounted together in the sensing chamber towards 50 ppm injection volumes of 1-butanol (d) 20% RH (e) 40% RH (f) 60% RH.

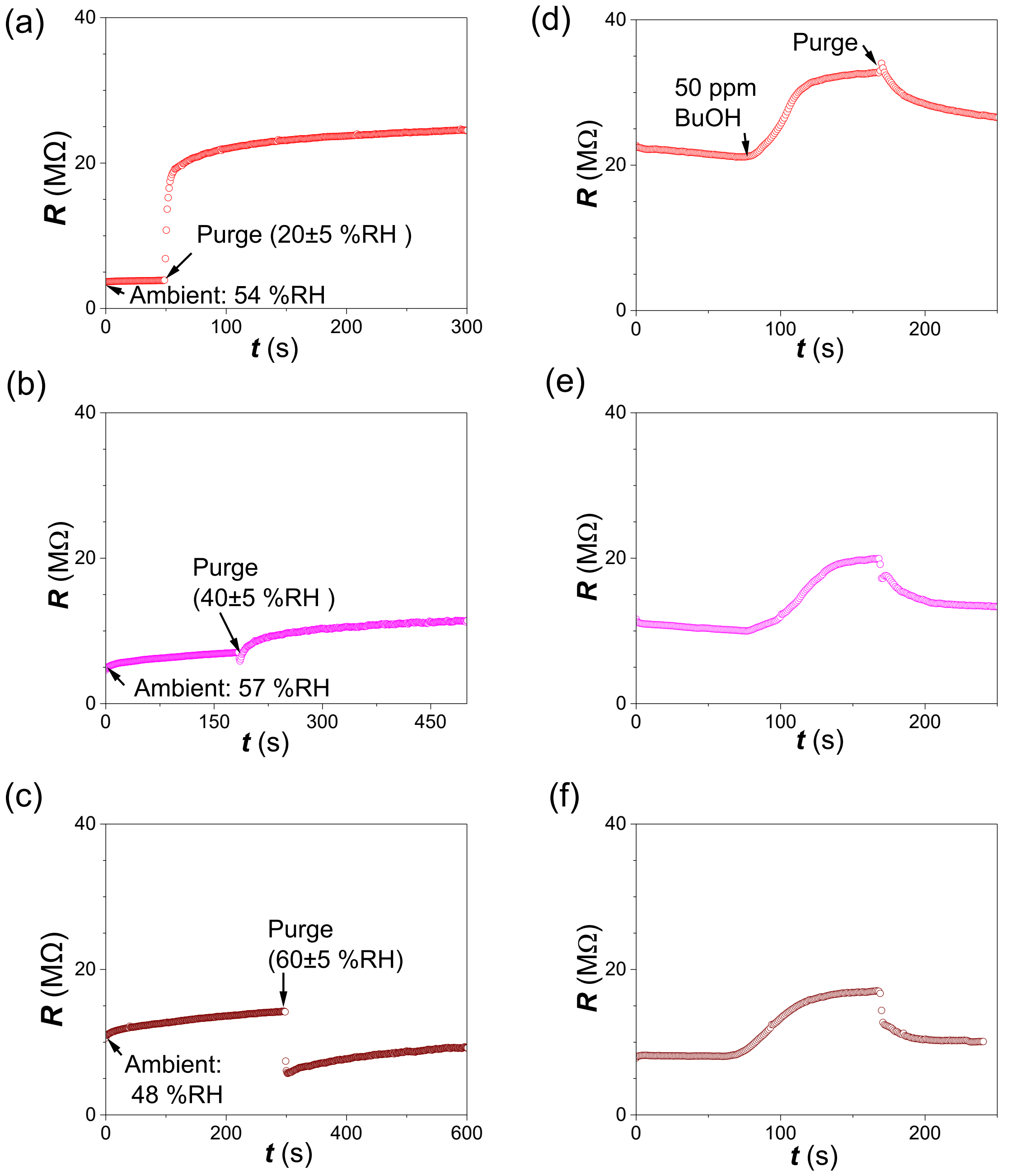

Figure S7. stabilization of the sensor through purging the humidified nitrogen to device mounted (D19) in the testing chamber to mimic desired humidity (a) 20% RH (b) 40% RH (c) 60% RH: Measured room-temperature resistance data vs time for α-Fe_2O_3 nanorod sensors mounted together in the sensing chamber towards 50 ppm injection volumes of 1-butanol (d) 20% RH (e) 40% RH (f) 60% RH.

Influence of carrier gas humidity on baseline resistance and response to 1-butanol

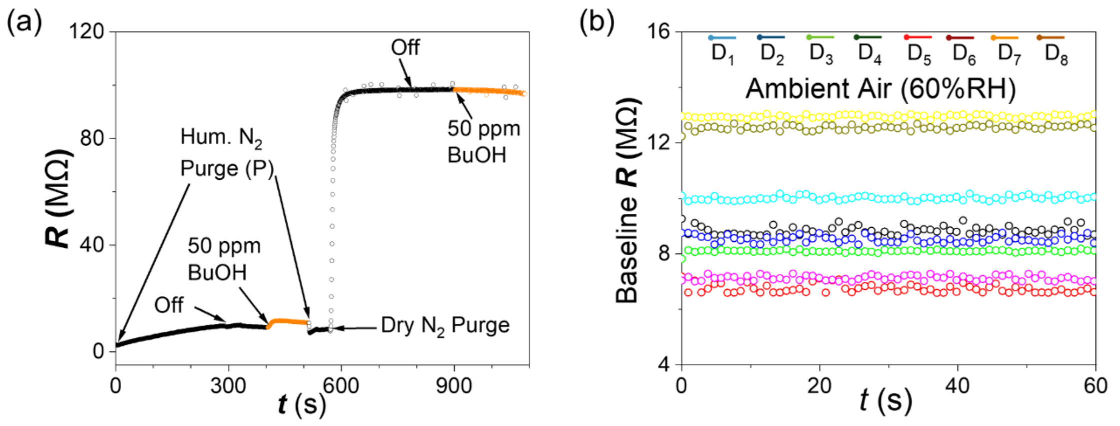

Figure S8. (a) Resistance of an α-Fe_2O_3 nanorod sensor (D21, measured simultaneously with D20) towards injection of 1 μL (~50 ppm) of 1-butanol followed by purging with humidified nitrogen (55±5 %RH) and dry nitrogen, respectively and injection of a second aliquot of 1-butanol. (b) Baseline resistance stability of α-Fe_2O_3 nanorod sensors (D1–D8) over time at room temperature under ambient conditions with 60% relative humidity.

VOC sensor performance (D1–D4)

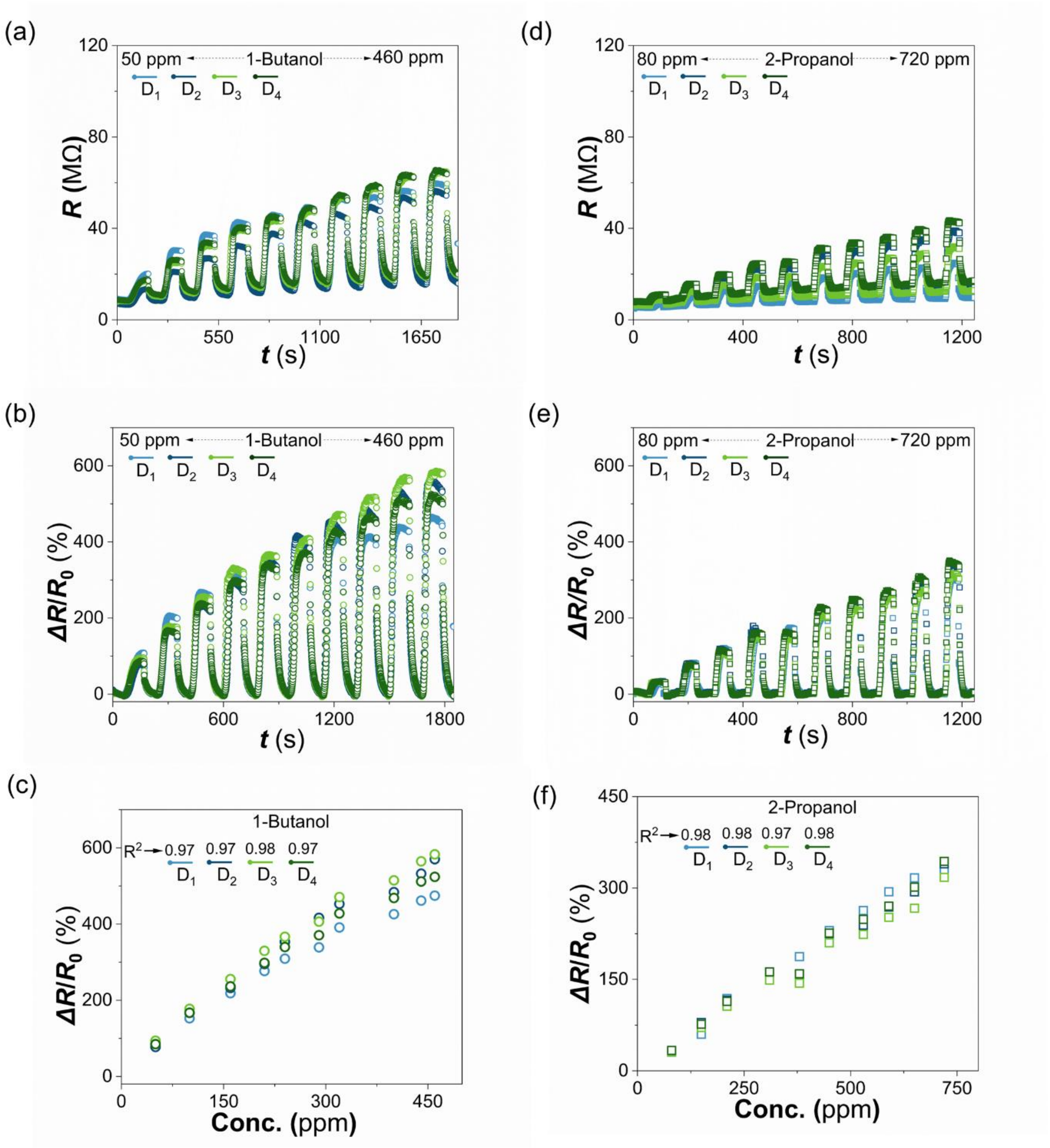

Figure S9. (a) Measured room-temperature resistance data vs time for four α-Fe_2O_3 nanorod sensors (D1-D4) mounted together in the sensing chamber towards a series of injected aliquots of 1-butanol from 1 mL to 10 μL. BuOH concentration taken as measured value after 3 minutes (Fig. S2a). (b) Normalised change in resistance (response), $\Delta R/R_0$ after baseline subtraction (c) Response vs concentration of 1-butanol (d) Resistance data for the same sensors towards increasing injection volumes of 2-propanol (IPA). IPA concentration taken as measured value after 3 minutes (Fig. S2b). (e) Normalised response, $\Delta R/R_0$, to IPA (f) Response vs concentration of IPA

Determination of Limit of Detection (*LOD*).

Table S3 Calculated *LOD* values from linear fits to device response, $\Delta R/R_0$ (%) vs VOC concentration (ppm)

(a) Devices exposed to 1-butanol injection series followed by 2-propanol injection series (D1 to D4) and devices exposed to 2-proposal injection series followed by 1-butanol injection series (D5-D8). Measured under humidified nitrogen (~60±5% RH).

**1-butanol**					**2-propanol**				
**Device**	***LOD*** (ppm)	**Error on Intercept**, σ_{int} (%)	**Slope, m_c** (%/ppm)	R^2	**Device**	***LOD*** (ppm)	**Error on Intercept**, σ_{int} (%)	**Slope, m_c** (%/ppm)	R^2
D1	32 ± 7	11.7	1.1	0.98	***D1***	52 ± 11	8.4	0.5	0.99
D2	21 ± 4	9.5	1.4	0.99	***D2***	60 ± 12	9.0	0.4	0.98
D3	29 ± 6	12.9	1.3	0.99	***D3***	65 ± 13	9.0	0.4	0.98
D4	33 ± 7	13.4	1.2	0.98	***D4***	54 ± 11	8.3	0.5	0.99
**Batch *LOD***	**29 ± 5**				**Batch *LOD***	**58 ± 6**			
D5	49 ± 11	20.1	1.2	0.96	***D5***	71 ± 15	9.2	0.4	0.98
D6	55 ± 12	20.1	1.1	0.96	***D6***	43 ± 9	6.9	0.5	0.99
D7	29 ± 6	11.3	1.2	0.99	***D7***	38 ± 8	5.6	0.4	0.99
D8	39 ± 8	15.9	1.2	0.98	***D8***	67 ± 14	9.6	0.4	0.98
**Batch *LOD***	**43 ± 11**				**Batch *LOD***	**55 ± 17**			

(b) Devices exposed to 1-butanol injection series under humidified nitrogen followed by 1-butanol injection series under humidified air (D24 to D27).

**1-butanol, humidified nitrogen (60±5% RH)**					**1-butanol, humidified air (60±5% RH)**				
**Device**	***LOD*** (ppm)	**Error on Intercept,** σ_{int} (%)	**Slope, m_c** (%/ppm)	R^2	**Device**	***LOD*** (ppm)	**Error on Intercept,** σ_{int} (%)	**Slope, m_c** (%/ppm)	R^2
D24	52 ± 12	18.9	1.1	0.97	***D24***	20 ± 4	10.9	1.6	1.00
D25	22 ± 5	8.4	1.2	0.99	***D25***	47 ± 10	19.0	1.2	0.97
D26	26 ± 5	13.1	1.5	0.99	***D26***	25 ± 5	11.7	1.4	0.99
D27	43 ± 9	18.3	1.3	0.98	***D27***	45 ± 10	17.4	1.2	0.98
**Batch *LOD***	**36 ± 14**				**Batch *LOD***	**34 ± 14**			

(c) Devices fabricated using α-Fe_2O_3 nanorods calcined at different temperatures (400–650°C) and measured under humidified nitrogen (~60±5% RH)

Device	T_{calc} (°C)	***LOD*** (ppm)	**Error on Intercept,** σ_{int} (%)	**Slope,** m_c (%/ppm)	R^2
D28	400	22 ± 5	0.63	0.08	0.99
D29	450	47 ± 10	1.46	0.09	0.97
D30	500	23 ± 5	4.54	0.60	0.99
D31	550	18 ± 4	7.00	1.15	1.00
D32	600	23 ± 5	18.54	2.46	0.99
D33	650	25 ± 5	20.80	2.50	0.99

Effect of repeated purging using humidified nitrogen

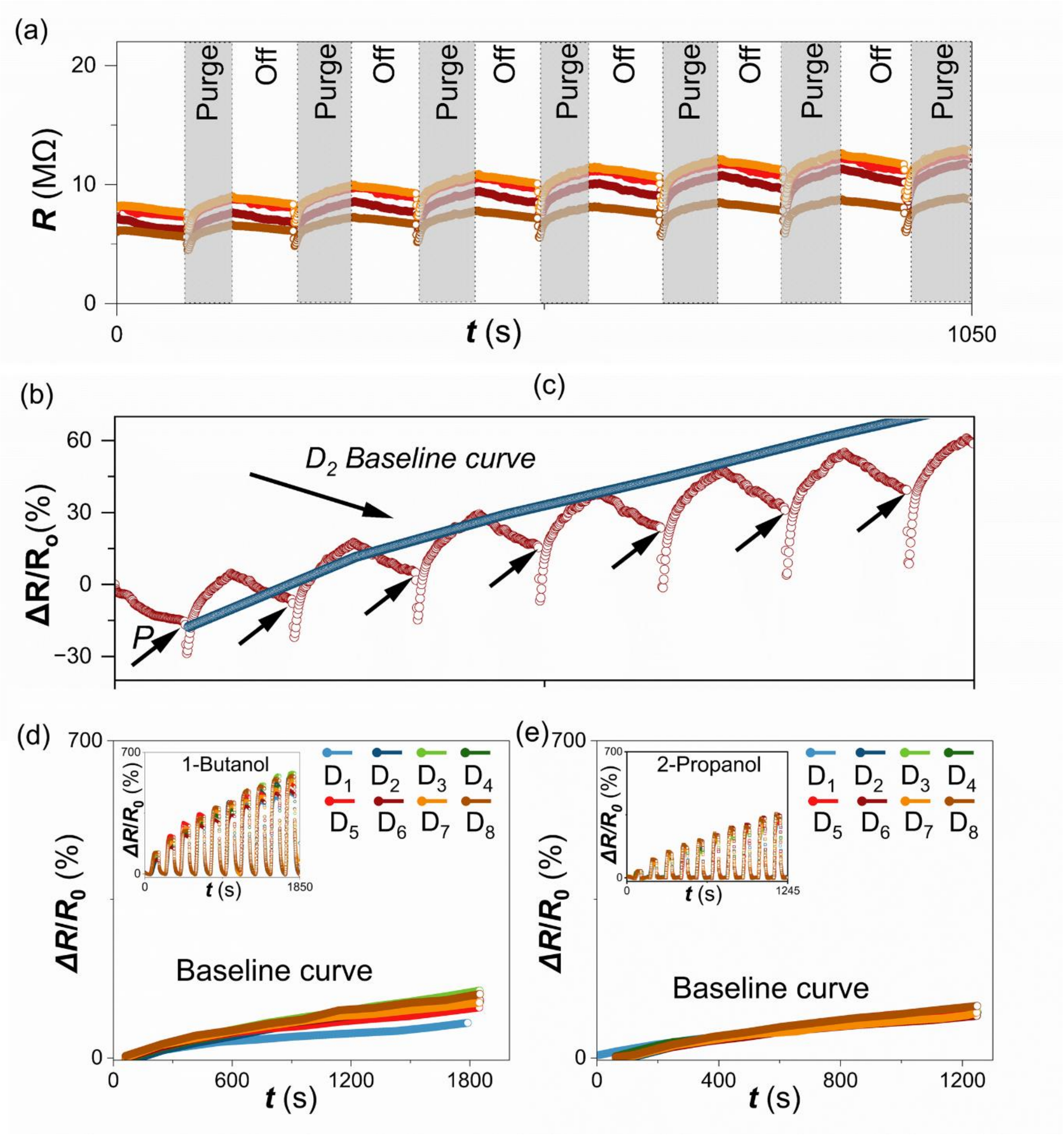

Figure S10. (a) Evolution of device resistance for four simultaneously measured α-Fe_2O_3 nanorod sensors (D9-D12) towards multiple cycles of purging with humidified nitrogen at relative humidity ~ 60%RH ("Purge"), followed by recovery period with humidified nitrogen line closed ("Off"). (b) Corresponding $\Delta R/R_0$ response for one device (D9) towards multiple cycles of purging with humidified nitrogen. Inset: Baseline curve for device D2. (d and e) show the calculated baseline curves for (D1-D8) for 1-butanol and 2- propanol measurements, respectively Insets: Baseline-subtracted $\Delta R/R_0$ data..

Sensor t_{90} response time constants following analyte injection.

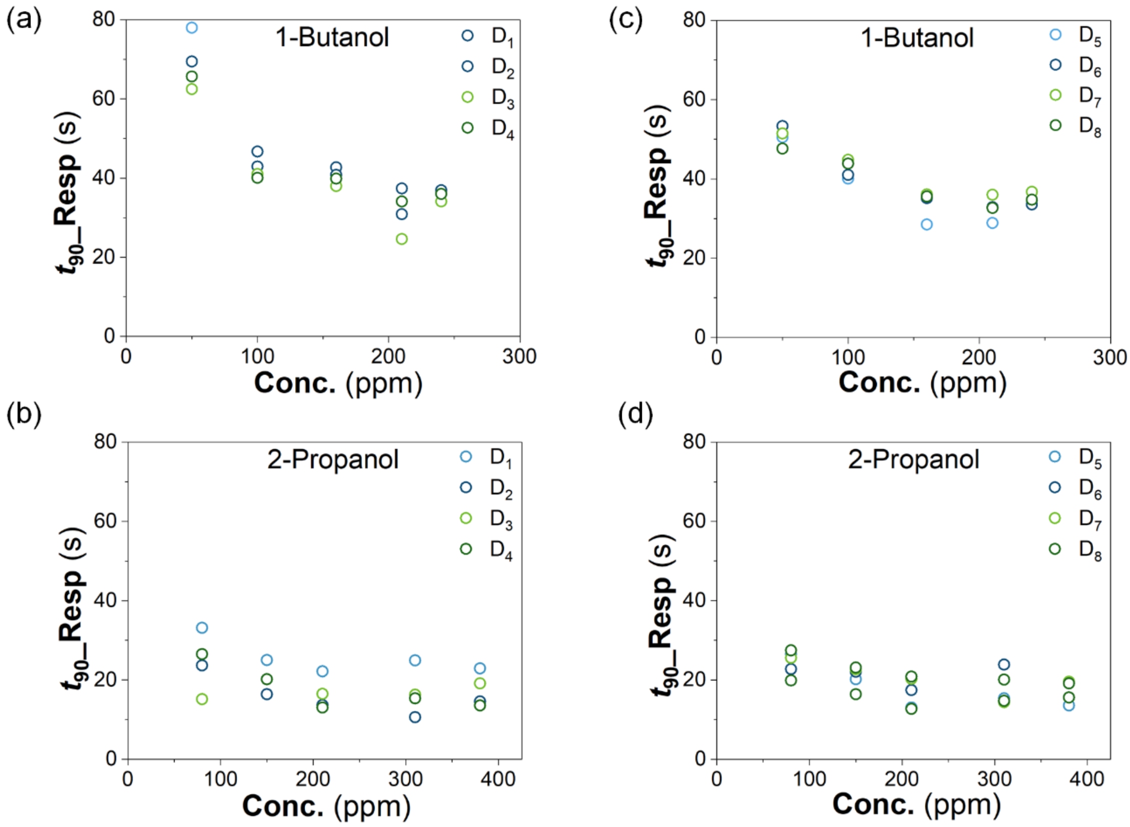

Figure S11. (a) Measured t_{90} response times for devices D1-D4 for each concentration of 1-butanol, calculated from $\Delta R/R_0$ data presented in Fig S3b. (b) Corresponding t_{90} response times for the same devices for 2-propanol (from $\Delta R/R_0$ data in Fig S3e). (c) 1-butanol t_{90} response times for D5-D8, calculated from $\Delta R/R_0$ data in Fig. S6e. (d) 2-propanol t_{90} response times for D5-D8, calculated from $\Delta R/R_0$ data in Fig. S6b.

VOC Sensing performance (D5–D8)

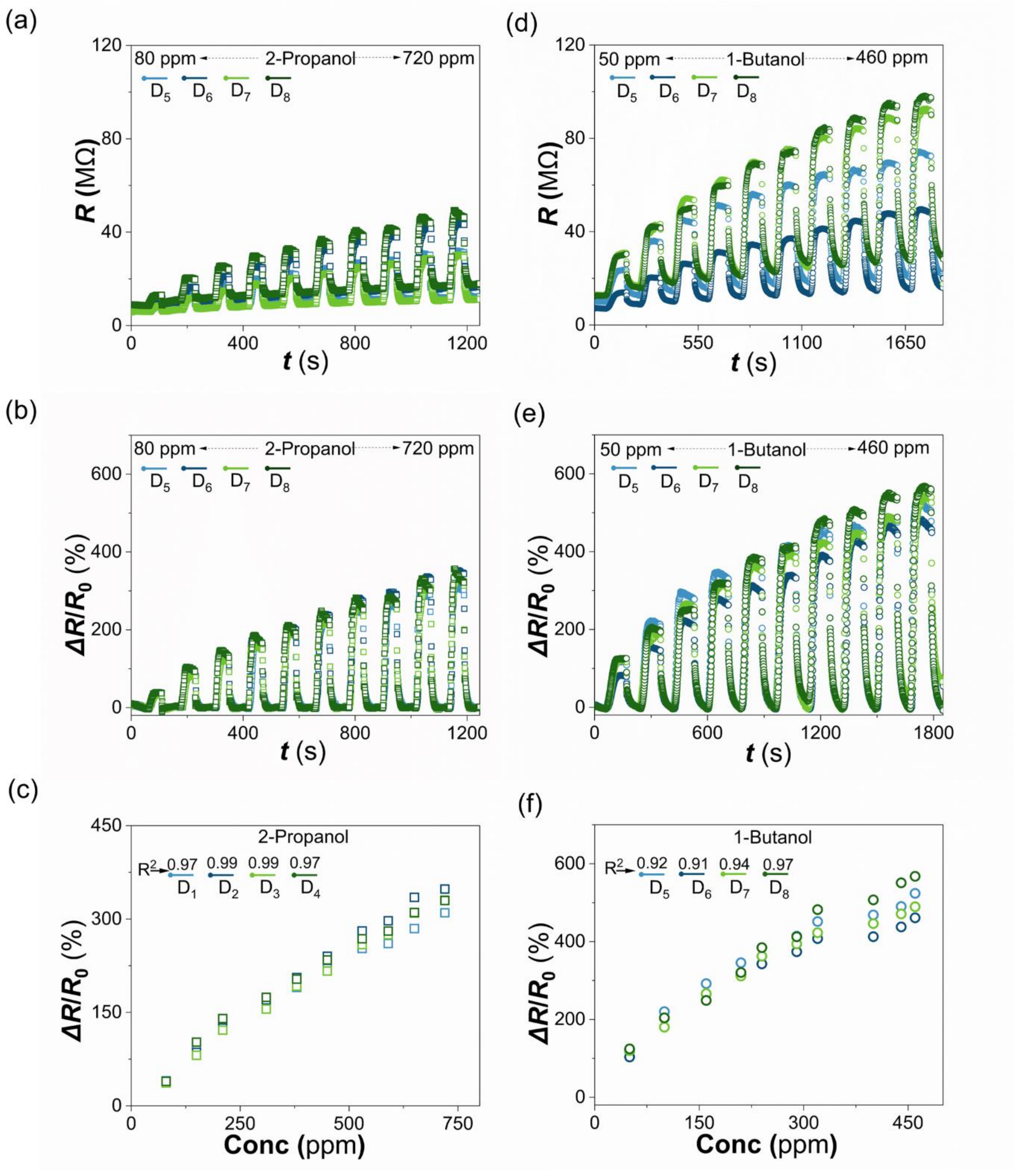

Figure S12. (a) Measured room-temperature resistance data vs time for four α-Fe_2O_3 nanorod sensors mounted together in the sensing chamber towards different injection volumes of 2-propanol (b) Normalised change in resistance (response), $\Delta R/R_0$ after baseline subtraction (Figure S5e) (c) Response vs concentration of 2-propanol for LOD calculation. (d) Resistance data for the same sensors towards increasing injection volumes of 1-butanol (e) $\Delta R/R_0$ after baseline subtraction (Figure S5d). (f) Response vs concentration of 1-butanol.

VOC sensing in humidified nitrogen vs humidified air

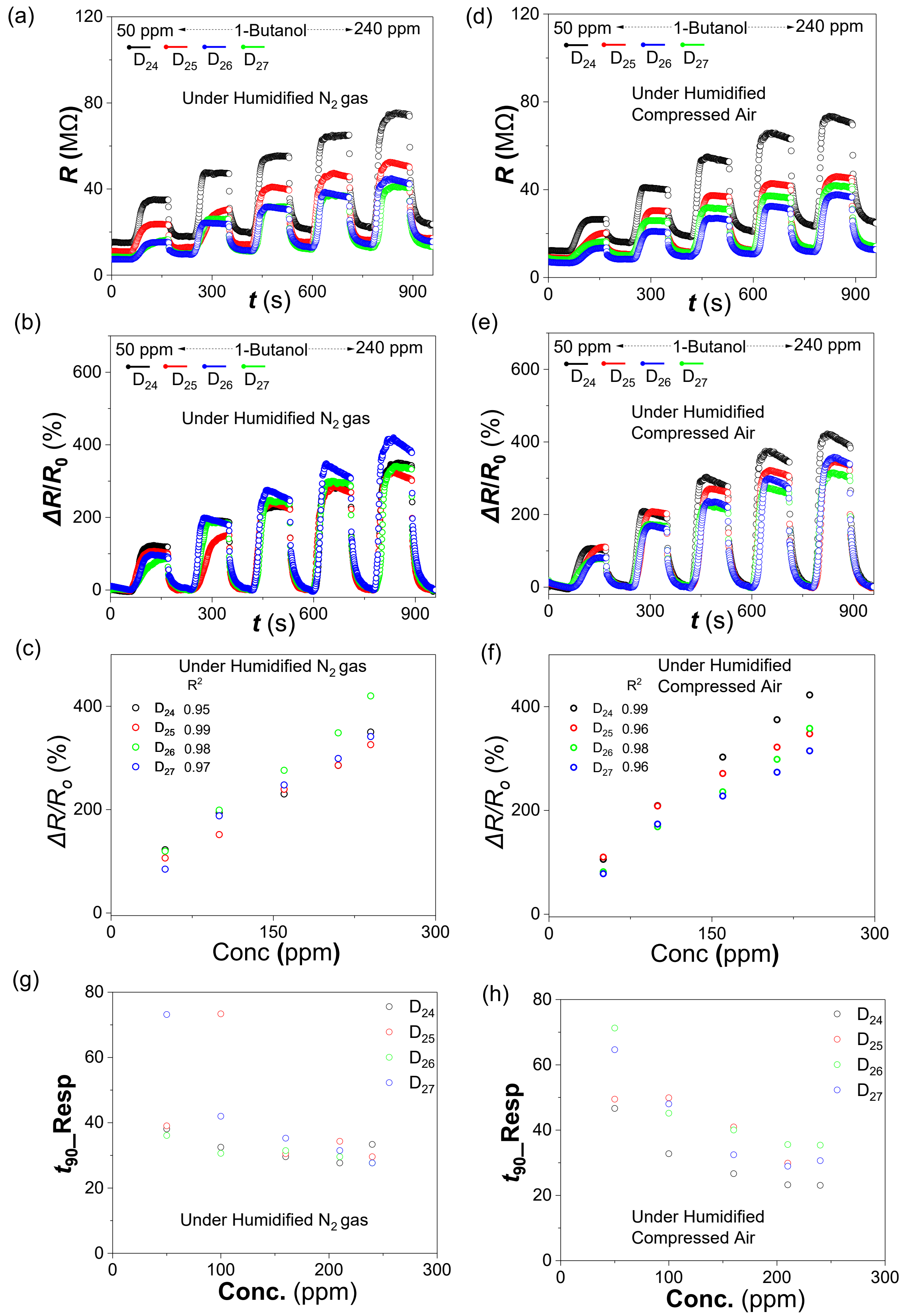

Figure S13. (a) Measured room-temperature resistance data vs time for four α-Fe_2O_3 nanorod sensors (D24-D27) mounted together in the sensing chamber towards a series of injected aliquots of 1-butanol from 1 mL to 5 μL under humidified nitrogen (~60±5% RH) with BuOH concentration taken as measured value after 3 minutes (Fig. S2a). (b) Normalised change in resistance (response), $\Delta R/R_0$ after baseline subtraction. (c) Response vs concentration of 1-butanol. (d) Resistance, (e) $\Delta R/R_0$ response, and (f), response vs concentration data for the same sensors measured under humidified air (~60±5% RH). (g) t_{90} response time constants extracted from response data (b) measured in humidified nitrogen. (h) t_{90} values from response data in humidified air (e).

Influence of calcination temperature, T_{calc}, on baseline resistance and response to 1-butanol.

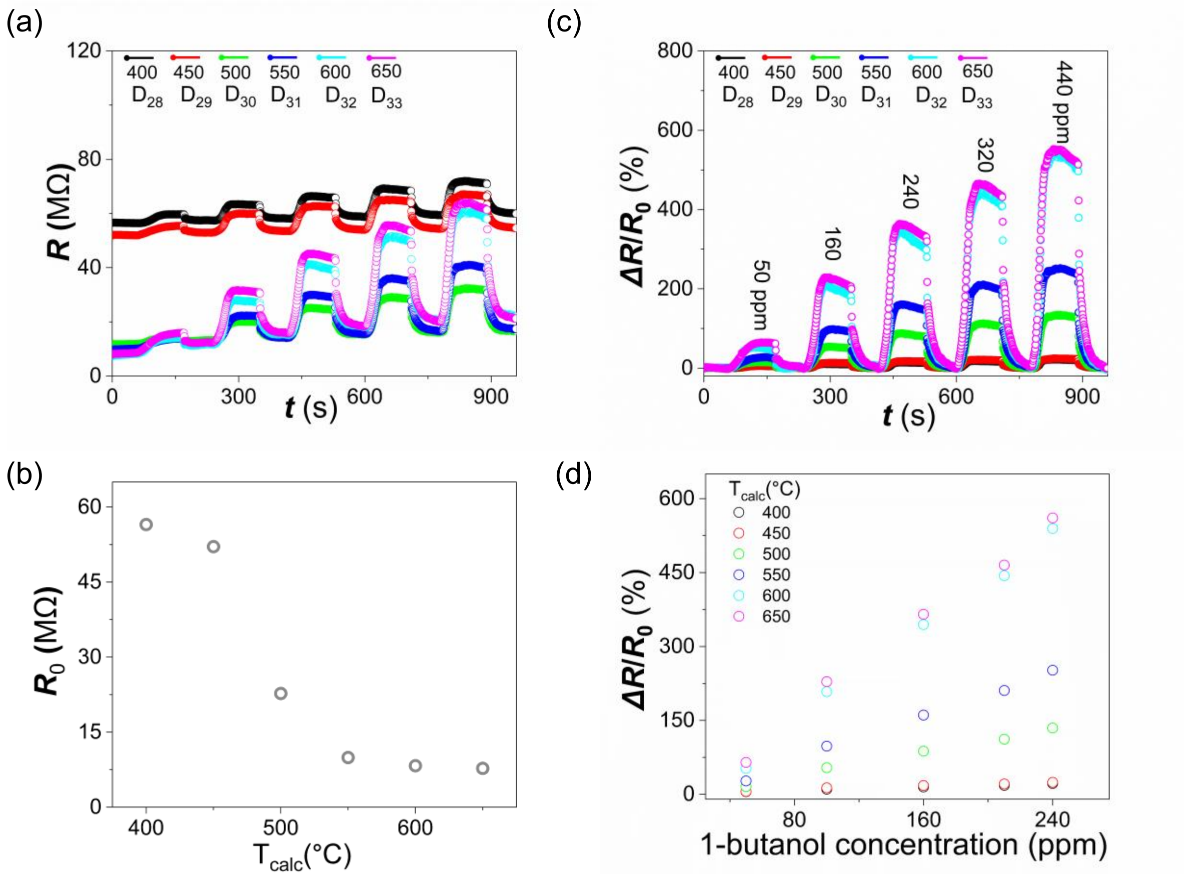

Figure S14. (a) Measured resistance (*R*) vs time (*t*) for a set of sensors (D_{28} – D_{33}), each fabricated from a batch of α-Fe_2O_3 nanorods calcined at a different temperature (T_{calc}) in the range 400–650°C. All devices were simultaneously exposed to a series of 1-butanol concentrations (50–240 ppm) at room temperature (20°C) and ~55% RH. (b) Baseline resistance values (R_0) for each sensor as a function of calcination temperature. (c) Baseline-corrected resistance response ($\Delta R/R_0$) vs time. (d) Peak response values across the tested concentration range (50–240 ppm) for each calcination temperature.

Table S3c shows the fit parameters and corresponding calculated *LOD* values. Five of the six devices show low *LOD* values, where the increase in the slope (m_c) is offset by an increase in the intercept error (σ_{int}).

Selectivity of LIG-contacted α-Fe_2O_3 nanorod devices

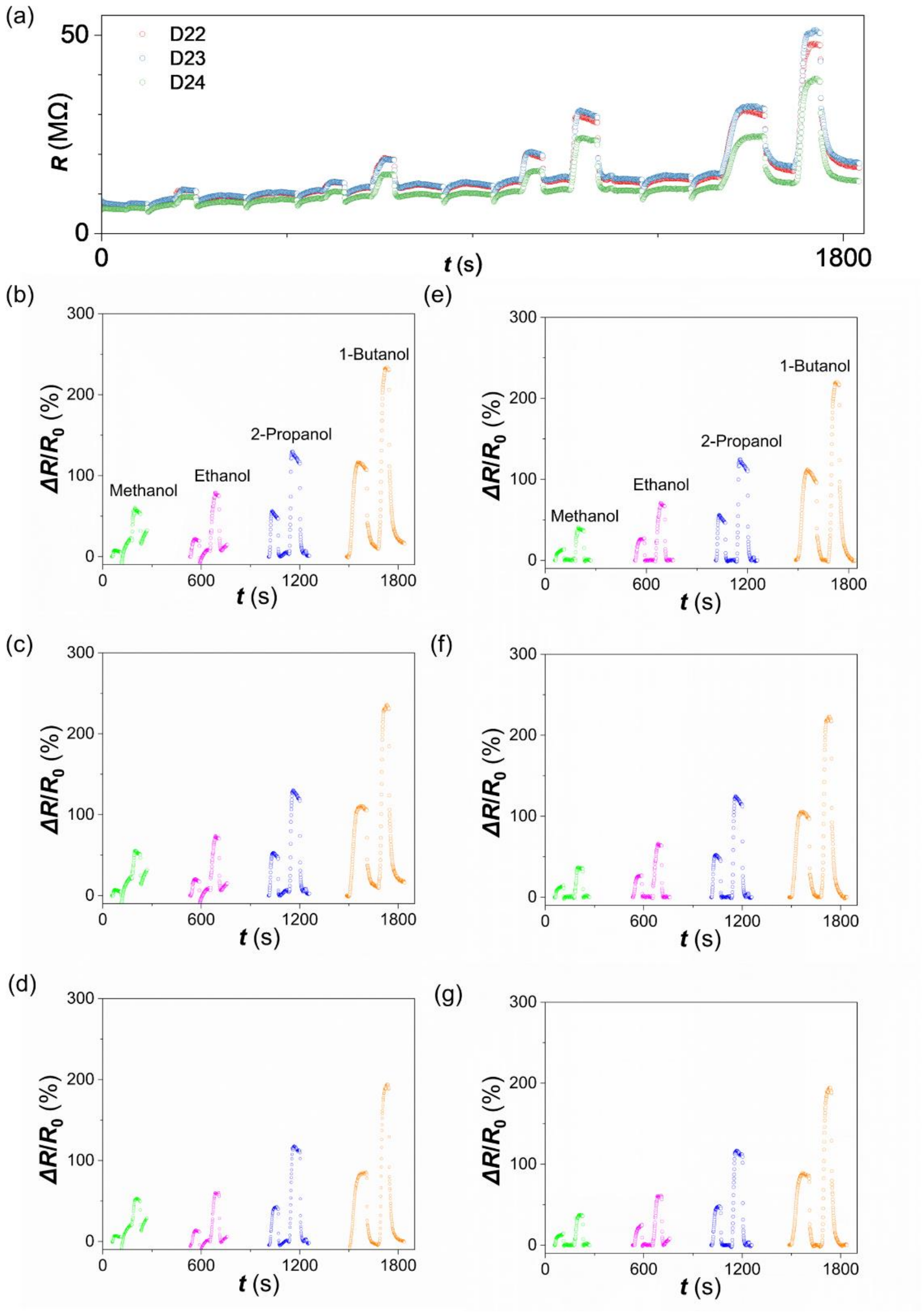

Figure S15. (a) Measured resistance vs time data for the VOC series with response data shown in Figure 5a. Corresponding $\Delta R/R_0$ response for the polar VOCs: (b) D22 (c) D23 (d) D24. (e,f,g) $\Delta R/R_0$ response following baseline curve subtraction.

Concentration-normalised response for each VOC

Table S4 Concentration-normalized response for each VOC, $\Delta R/R_{0,100\ ppm}$

				**$\Delta R/R_o$* per 100 ppm (%)**					
**Conc (μL)**	**Analyte**	**#C atoms**	**Conc (ppm)**	**D22**	**D23**	**D24**	**Mean μ (%)**	**StDev, σ (%)**	**CoV, σ / μ**
1	MeOH	1	150	10	10	10	10	0.2	0.02
3	MeOH	1	430	9	9	9	9	0.4	0.04
1	EtOH	2	90	29	30	28	29	1.1	0.04
3	EtOH	2	170	41	39	36	39	2.8	0.07
1	IPA	3	80	70	65	59	65	5.5	0.08
3	IPA	3	210	60	60	56	58	2.2	0.04
1	BuOH	4	50	224	209	175	203	25.4	0.13
3	BuOH	4	160	137	139	122	133	9.8	0.07

Comparison of $\Delta R/R_0$ response selectivity vs literature

These LIG/a-Fe_2O_3 devices also show good selectivity when compared to other chemi-resistive sensors targeting detection of 1-butanol.(31) Defining a response magnitude selectivity ratio for 1-butanol vs a given VOC ($SR_{\mathrm{BuOH}:VOC}$) as

$$SR_{\mathrm{BuOH:}VOC} = \left[\frac{\Delta R}{R_0}\right]_{BuOH@100ppm} \Big/ \left[\frac{\Delta R}{R_0}\right]_{VOC,@100ppm} \quad (2)$$

yields $SR_{\mathrm{BuOH}:VOC}$ results that compare well with values calculated from reported data for room temperature sensing using MOF-derived Fe_2O_3 nanocubes and Fe_2O_3 nanocubes combined with resource- intensive reduced graphene oxide [15]; and elevated temperature sensing (140°C) using MOF-derived synthesis of Co_3O_4 nanospheres[16];see Table S5.

Table S5 VOC Response Magnitude Selectivity Comparison vs 1-butanol

**Sensing material**	***SR*VOC**					**Ref.**
	**Methanol**	**Ethanol**	**IPA**	**Acetone**	**Toluene**	
Co_3O_4 nanospheres	2	2	2.5	2.5	1.5	[16]
α-Fe_2O_3 nano cubes/rGO	17	11.5	*N/A*	14.5	*N/A*	[15]
α-Fe_2O_3 nano cubes	5	3	*N/A*	4	*N/A*	[15]
α-Fe_2O_3 nanorods	15±1	3.4±0.1	2.3±0.05	>50	>50	This work

I-V measurements in different VOC environments

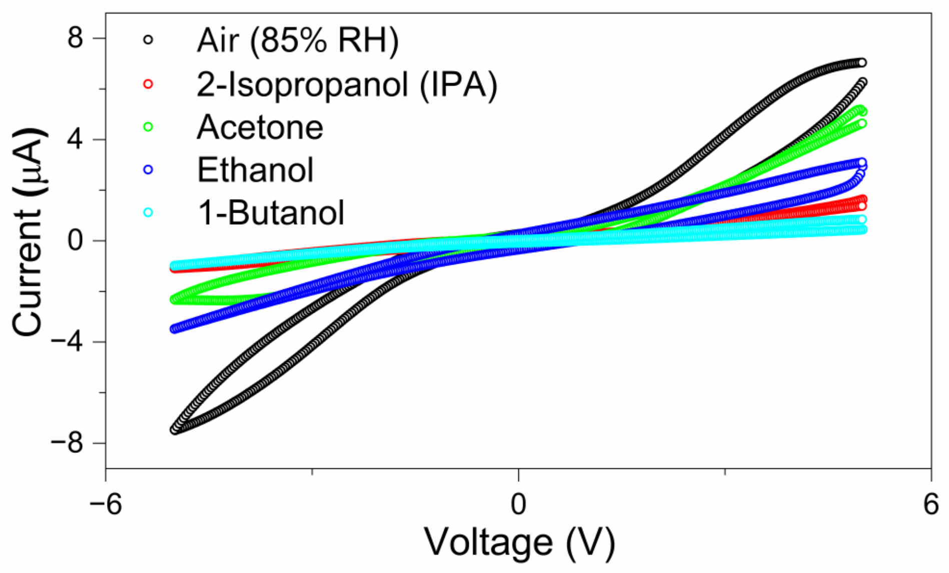

Figure S16. (a) Current voltage (*I-V*) measurements of a LIG-contacted α-Fe_2O_3 nanorod device mounted in a series of 5 centrifuge tubes (50 mL volume) , each containing a different vapor environment (i) Humidity standard (KCL, 85% RH), (ii) 5 μL IPA), (iii) 5 μL acetone, (iv) 5 μL ethanol, (v) 5 μL 1-butanol. For each environment, the device was allowed to stabilise in each tube for ~30 minutes before the *I-V* measurement (5 V → -5 V→ 5 V) and then the device was left in ambient air for ~24 hours to recover.

Machine Learning Models for concentration-normalised resistance response and t_{90} times

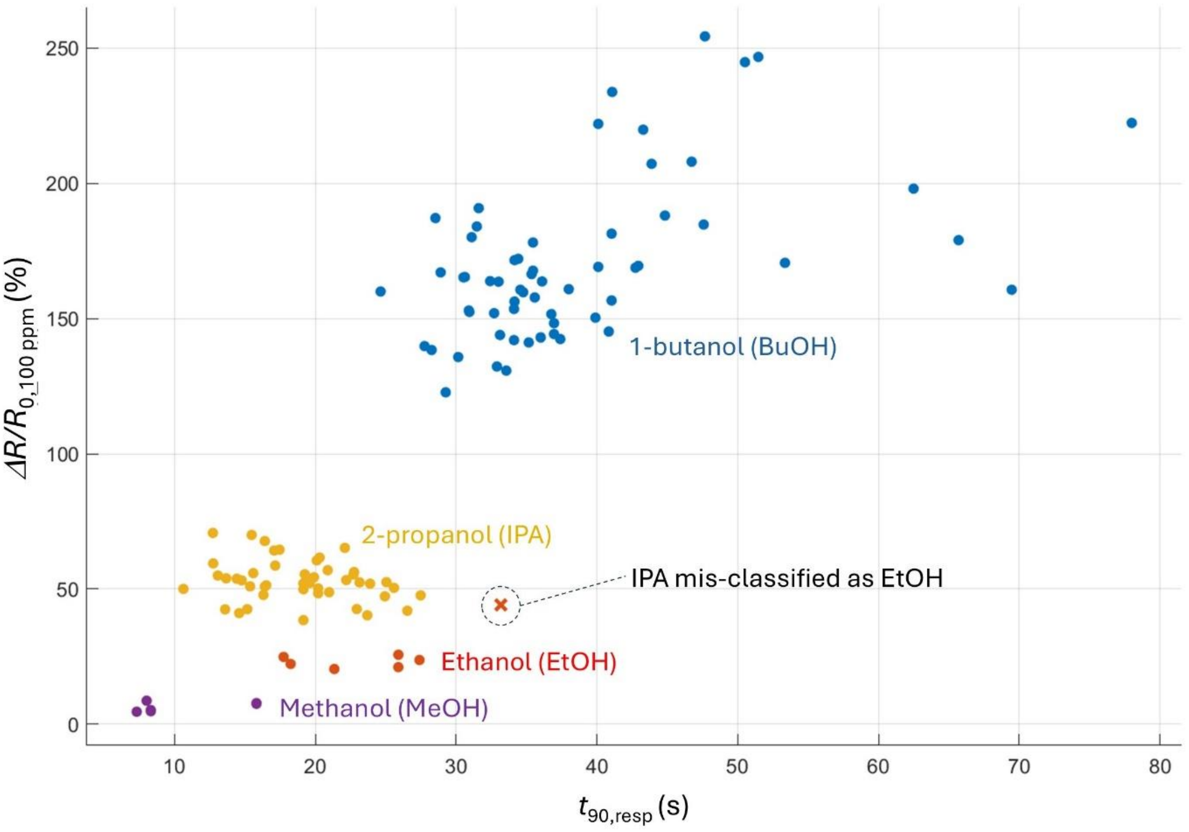

Figure S17. MATLAB fine-grained KNN model classification of concentration-normalized response data $\Delta R/R_{0,100\ ppm}$; t_{90} response time constant after VOC injection, $t_{90,resp}$; and t_{90} recovery time constant after purging with humidified nitrogen, $t_{90,rec}$. Dashed circle shows 2-propanol outlier misclassified as ethanol. Datasets provided below in Tables S6-S9.

Table S6 Model input data for 1-butanol (BuOH)

Analyte	Device	Conc. (ppm)	t_resp (s)	t_rec (s)	Resp_100ppm (%)	Comment
BuOH	D1	50	78	31	222	
BuOH	D1	100	47	24	208	
BuOH	D1	160	43	24	169	
BuOH	D1	210	31	23	153	
BuOH	D1	240	37	25	144	
BuOH	D2	50	69	30	161	
BuOH	D2	100	43	22	169	
BuOH	D2	160	41	25	145	
BuOH	D2	210	37	23	142	
BuOH	D2	240	37	24	148	
BuOH	D3	50	62	30	198	
BuOH	D3	100	41	24	181	
BuOH	D3	160	38	23	161	
BuOH	D3	210	25	23	160	
BuOH	D3	240	34	25	154	
BuOH	D4	50	66	44	179	
BuOH	D4	100	40	33	169	
BuOH	D4	160	40	29	150	
BuOH	D4	210	34	28	142	
BuOH	D4	240	36	29	143	
BuOH	D5	50	51	27	245	
BuOH	D5	100	40	26	222	
BuOH	D5	160	29	27	187	
BuOH	D5	210	29	24	167	
BuOH	D5	240	35	25	161	
BuOH	D6	50	53	24	171	
BuOH	D6	100	41	24	157	
BuOH	D6	160	35	26	141	
BuOH	D6	210	33	23	132	
BuOH	D6	240	34	23	131	
BuOH	D7	50	51	25	247	
BuOH	D7	100	45	26	188	
BuOH	D7	160	36	25	164	
BuOH	D7	210	36	26	143	
BuOH	D7	240	37	25	152	
BuOH	D8	50	48	24	254	
BuOH	D8	100	44	25	207	
BuOH	D8	160	36	26	158	
BuOH	D8	210	33	23	152	
BuOH	D8	240	35	24	160	
BuOH	D13	50	32	4	164	
BuOH	D13	50	35	5	168	
BuOH	D13	50	35	4	166	
BuOH	D13	50	31	4	184	
BuOH	D14	50	30	3	136	

Analyte	Device	Conc. (ppm)	t_resp (s)	t_rec (s)	Resp_100ppm (%)	Comment
BuOH	D14	50	33	4	144	
BuOH	D14	50	31	3	152	
BuOH	D14	50	31	4	165	
BuOH	D15	50	34	2	156	
BuOH	D15	50	33	3	164	
BuOH	D15	50	31	3	165	
BuOH	D15	50	31	3	180	
BuOH	D16	50	34	3	172	
BuOH	D16	50	35	3	178	
BuOH	D16	50	34	4	172	
BuOH	D16	50	32	4	191	
BuOH	D22	50	41	26	234	
BuOH	D23	50	43	19	220	
BuOH	D24	50	48	18	185	
BuOH	D22	160	28	28	138	
BuOH	D23	160	28	25	140	
BuOH	D24	160	29	23	123	

Table S7 Model input data for 2-propanol (isopropyl alcohol, IPA)

Analyte	Device	Conc (ppm)	t_resp (s)	t_rec (s)	Resp_100ppm (%)	Comment
IPA	D1	80	33	10	44	*
IPA	D1	150	25	10	52	
IPA	D1	210	22	10	53	
IPA	D1	310	25	9	47	
IPA	D1	380	23	11	43	
IPA	D2	80	24	17	40	
IPA	D2	150	16	6	51	
IPA	D2	210	14	8	54	
IPA	D2	310	11	10	50	
IPA	D2	380	15	9	41	
IPA	D3	80	15	27	43	
IPA	D3	150	20	8	48	
IPA	D3	210	17	10	51	
IPA	D3	310	16	11	48	
IPA	D3	380	19	11	38	
IPA	D4	80	27	27	42	
IPA	D4	150	20	7	50	
IPA	D4	210	13	9	55	
IPA	D4	310	15	10	51	
IPA	D4	380	14	9	42	
IPA	D5	80	23	11	56	*
IPA	D5	150	22	11	65	
IPA	D5	210	17	11	65	
IPA	D5	310	24	11	52	
IPA	D5	380	19	11	52	
IPA	D6	80	26	5	50	
IPA	D6	150	23	11	55	
IPA	D6	210	20	9	62	
IPA	D6	310	14	10	54	
IPA	D6	380	20	10	52	
IPA	D7	80	27	5	48	
IPA	D7	150	23	10	52	
IPA	D7	210	21	9	57	
IPA	D7	310	15	10	53	
IPA	D7	380	19	9	50	
IPA	D8	80	20	5	54	
IPA	D8	150	16	8	68	
IPA	D8	210	13	9	71	
IPA	D8	310	20	9	61	
IPA	D8	380	16	9	56	
IPA	D22	80	15	2	70	
IPA	D23	80	17	3	64	
IPA	D24	80	21	2	49	
IPA	D22	210	13	11	59	
IPA	D23	210	17	10	59	

Analyte	Device	Conc (ppm)	t_resp (s)	t_rec (s)	Resp_100ppm (%)	Comment
IPA	D24	210	19	12	55	

* Noisy signal for this trace, recovery time was estimated as average of recovery times for other IPA concentrations for this device run.

Table S8 Model input data for ethanol (EtOH)

Analyte	Device	Conc(ppm)	t90_resp(s)	t90_rec(s)	Resp_100ppm(%)	Comment
EtOH	D22	90	18	3	25	
EtOH	D23	90	18	4	22	
EtOH	D24	90	21	5	20	
EtOH	D22	280	26	9	26	
EtOH	D23	280	27	8	24	
EtOH	D24	280	26	9	21	

Table S9 Model input data for methanol (MeOH)

Analyte	Device	Conc(ppm)	t90_resp(s)	t90_rec(s)	Resp_100ppm(%)	Comment
MeOH	D22	150	7	4	5	
MeOH	D23	150	8	4	5	
MeOH	D24	150	8	6	5	
MeOH	D22	430	8	7	9	
MeOH	D23	430	16	6	8	
MeOH	D24	430	16	6	8	

Device summary

Table S10: Devices studied (D1–D33) with key metrics. $\Delta R/R_0$ response values for 1-butanol at room temperature, while selectivity is based on normalized VOC responses. RH indicates chamber humidity during sensing. Values are means ± SD, with 'n' showing device number.

Devices	Purpose	Figure(s) & Table(s)	Carrier Gas	RH (%)	$\Delta R/R_0$ 1-BuOH (%)	LOD 1-BuOH (ppm)	Comments
**D1-D8**	Response/sensitivity	SI Fig. S9; SI Fig. S12; SI Table S3a; SI Fig. S7	Humidified N_2	60±5	100 ± 18 (n=8), at 50 ppm	35±10 (per-device, 50–300 ppm)	Linear dynamic range 50-300 ppm, 41 ± 11 t_{90} resp(s)
**D9-D12**	Baseline drift	SI Fig. S5	Humidified N_2	60±5	-	-	Purge-only; Response drift ≈ 55 ± 7% (n=4)
**D13-D16**	Reproducibility at 50 ppm	Main Fig. 5a-b; SI Fig. S8	Humidified N_2	60±5	83 ± 1 (n=4, 16 analyte cycles), at 50 ppm	-	Cycle-to-cycle CoV 0.05–0.08
**D17**	RH calibration	Main Fig. 3a; SI Fig. S5-S18b	Salt standards	7.5–85	-	-	quasi-logarithmic dependence vs RH
**D18-D19**	RH dependence of response to 50 ppm BuOH	SI Fig. S11; SI Fig. S12	Humidified N_2	~20/40/60	112 ± 4 (60%RH); 97 ± 2 (40%RH); 53 ± 2 (20%RH)	-	Humidity assisted sensing mechanism
**D20-D21**	Humidity influence	Main Fig. 3b; SI Fig. S13a	N_2 (humid vs dry)	60±5	12.52 to 18.23MΩ at 60%RH<br>Dry: ~99MΩ	-	corroborated with Humidity assisted sensing mechanism
**D22-D23**	Selectivity	Main Fig. 5a-b; SI Fig. S15; SI Table S4	Humidified N_2	60±5	133 ± 9.8 per 100 ppm	-	EtOH: 39 ± 2.8; IPA: 58 ± 2.2; MeOH: 9 ± 0.4 (per 100 ppm)
**D24-D27**	Humidified N_2 vs Humidified Air (carrier gas comparison)	SI Fig. S13; SI Table S3b	Humidified N_2 Humidified Air	60±5	N_2: 183.04 ± 21.43; Air: 190.01 ± 21.96 (at 100 ppm)	N_2: 36 ± 14; Air: 34 ± 14	Comparable responses, response time and LODs.
**D28**	T_{calc}: 400 °C	SI Fig. S14; SI Table S3c	Humidified N_2	60±5	10% at 100 ppm	22 ± 5	Weak **$\Delta R/R_0$** response
**D29**	T_{calc}: 450 °C	SI Fig. S14; SI Table S3c	Humidified N_2	60±5	13% at 100 ppm	47 ± 10	-
**D30**	T_{calc}: 500 °C	SI Fig. S14; SI Table S3c	Humidified N_2	60±5	54% at 100 ppm	23 ± 5	-
**D31**	T_{calc}: 550 °C	SI Fig. S14; SI Table S3c	Humidified N_2	60±5	98% at 100 ppm	18 ± 4	-
**D32**	T_{calc}: 600 °C	SI Fig. S14; SI Table S3c	Humidified N_2	60±5	208% at 100 ppm	23 ± 5	Large **$\Delta R/R_0$** response increase
**D33**	T_{calc}: 650 °C	SI Fig. S14; SI Table S3c	Humidified N_2	60±5	228% at 100 ppm	25 ± 5	Large response magnitude

UV-Vis absorption spectrum and Tauc plot for α-Fe_2O_3 nanorod solution

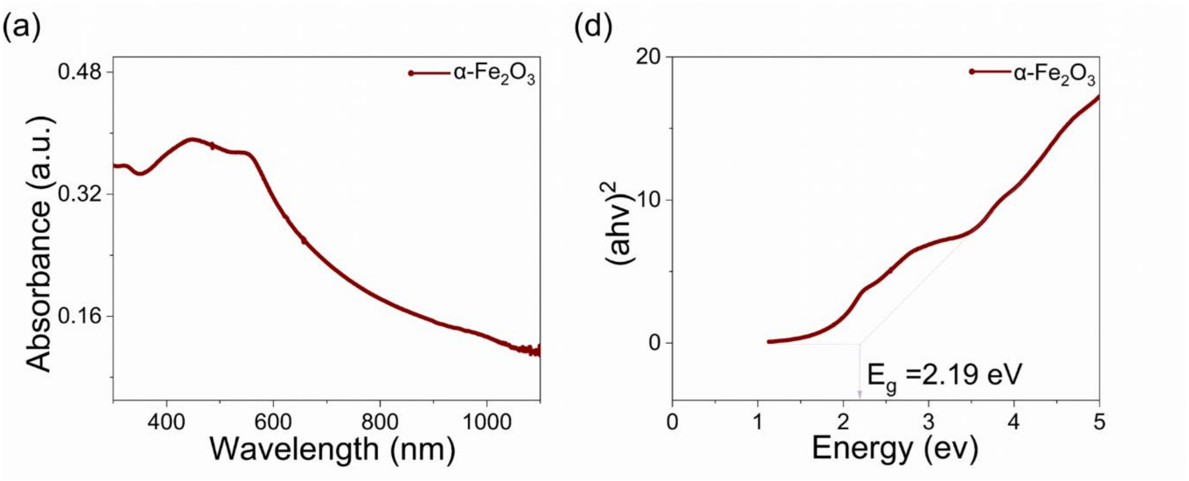

Figure S18. (a) UV-Vis Absorbance spectrum of α-Fe_2O_3 nanorod solution (b) Corresponding Tauc plot

SI References

1 H. Pan, L. Jin, B. Zhang, H. Su, H. Zhang and W. Yang, *Sens Actuators B Chem*, 2017, **243**, 29–35.

2 M. Li, B. Li, F. Meng, J. Liu, Z. Yuan, C. Wang and J. Liu, *Sens Actuators B Chem*, 2018, **273**, 543–551.

3 Z. Zhao, D. Jiang, Y. Xue, Y. Sun, W. L. Zhang, P. Li, D. Long, W. Gong and J. Hu, *J Mater Sci*, 2021, **56**, 16963–16975.

4 M. Wang, T. Hou, Z. Shen, X. Zhao and H. Ji, *Sens Actuators B Chem*, 2019, **292**, 171–179.

5 Y. V. Kaneti, Q. M. D. Zakaria, Z. Zhang, C. Chen, J. Yue, M. Liu, X. Jiang and A. Yu, *J Mater Chem A Mater*, 2014, **2**, 13283–13292.

6 Y. Xu, X. Tian, D. Sun, Y. Sun and D. Gao, *Z Anorg Allg Chem*, 2019, **645**, 447–456.

7 Y. Li, X. Wang, G. Sun, J. Cao and Y. Wang, *Vacuum*, 2023, **216**, 112478.

8 M. Wang, J. Shao, H. Liu, Y. Qi, P. He, S. Yue, C. Sun, J. Dong, G. Pan and X. Yang, *ACS Appl Mater Interfaces*, 2022, **15**, 9862–9872.

9 Q. Luo, J. Wu, S. Zou, W. Wang, Z. Wang, Y. Wan and C. Feng, *J Taiwan Inst Chem Eng*, 2023, **145**, 104820.

10 R. Mo, D. Han, C. Yang, J. Tang, F. Wang and C. Li, *Sens Actuators B Chem*, 2021, **330**, 129326.

11 G. Korotcenkov, 2008, preprint, DOI: 10.1016/j.mser.2008.02.001.

12 X. Li, D. Li, J. Xu, H. Jin, D. Jin, X. Peng, B. Hong, J. Li, Y. Yang, H. Ge and X. Wang, *Powder Technol*, 2017, **318**, 40–45.

13 Z. Jin, H.-J. Zhou, Z.-L. Jin, R. F. Savinell and C.-C. Liu, *Application of nano-crystalline porous tin oxide thin film for CO sensing*, 1998, vol. 52.

14 M. N. Rumyantseva, A. M. Gaskov, N. Rosman, T. Pagnier and J. R. Morante, *Chemistry of Materials*, 2005, **17**, 893–901.

15 R. Mo, D. Han, C. Yang, J. Tang, F. Wang and C. Li, *Sens Actuators B Chem*, 2021, **330**, 129326.

16 L. Cheng, Y. He, M. Gong, X. He, Z. Ning, H. Yu and Z. Jiao, *J Alloys Compd*, 2021, **857**, 158205.
